# Reframing glucolipid metabolic disorders through the lens of the oral microbiome: from pathophysiological mechanisms to translational potential

**DOI:** 10.3389/fnut.2026.1819765

**Published:** 2026-07-10

**Authors:** Pingjie Xiong, Bin Wang, Yufei Liu, Zhihui Wang, Xiaoqiang Huang, Jiao Guo

**Affiliations:** 1 Guangdong Metabolic Diseases Research Center of Integrated Chinese and Western Medicine, Guangdong Pharmaceutical University, Guangzhou, China; 2 Key Laboratory of Glucolipid Metabolic Disorder, Ministry of Education, Guangdong Pharmaceutical University, Guangzhou, China; 3 Guangdong TCM Key Laboratory for Metabolic Diseases, Guangdong Pharmaceutical University, Guangzhou, China; 4 Institute of Chinese Medicine, Guangdong Pharmaceutical University, Guangzhou, China; 5 The First Affiliated Hospital of Guangdong Pharmaceutical University, Guangdong, China

**Keywords:** disease prediction, glucolipid metabolic disorders (GLMD), mechanisms, metabolic comorbidities, microecological dysbiosis, oral microbiome

## Abstract

With the rising prevalence of metabolic diseases, their comorbidity is increasingly common. Glucolipid metabolic disorders (GLMD) constitute a major health challenge associated with increased cardiovascular risk and mortality. Despite many advances in disease mechanisms and therapeutic strategies, the metabolic disease burden remains substantial. Routine clinical practice still lacks straightforward approaches for identifying individuals at elevated risk, and the challenge of effectively preventing and controlling GLMD has become an urgent clinical problem. Significant gaps remain in our understanding of metabolic disease. Advances in high-throughput sequencing and omics technologies have expanded the research perspective, shifting attention from organ-centered pathology toward microecological and metabolic networks, elucidating the interactions between the oral microbiota and host metabolism. As the second largest microbial community after the gut microbiota, the oral microbiome provides an integrative lens for examining GLMD-related microbial signatures, plausible mechanistic pathways, and translational opportunities, including inflammation, taste signaling, nitric oxide metabolism, and microbial translocation. This review aims to clarify the relationship between the oral microbiome and GLMD, outline characteristic microbial alterations in metabolic diseases, and summarize the plausible mechanisms linking oral microorganisms with metabolic homeostasis. Current evidence indicates partially recurrent microbial patterns across GLMD-related conditions, but no universal oral microbial signature has been established. Building on recent findings, we conclude by outlining the methodological requirements for evaluating the clinical utility of oral microbiome-based markers and interventions, and further discuss the value of oral microbiota in disease prediction, risk assessment, and individualized intervention. We also summarize the key limitations and challenges in the field and outline directions for future prevention and control of GLMD based on oral microecology.

## 1 Introduction

Metabolic diseases have a high comorbidity rate, pose substantial health risks, and show a progressively worsening trend. As reported by The Lancet, the greatest cumulative threat to global health arises from the ongoing rise in metabolic risk factors (1). Moreover, patients with comorbid glucolipid metabolic diseases generally have higher risks of cardiovascular events and all-cause mortality than those with a single metabolic disease (2, 3). Glucolipid metabolic disorders (GLMD) is a complex disease influenced by genetic, environmental, psychological, and dietary factors. It is characterized by disturbances in glucose and lipid metabolism. Clinically, GLMD manifests primarily as hyperglycemia, dyslipidemia, hepatic steatosis (4). Therefore, understanding GLMD requires an integrated perspective that considers both individual metabolic diseases and their shared pathological backgrounds, which is essential for improving prevention and comprehensive management strategies.

Advances in high-throughput and omics technologies have shed light on the complex interactions between host systems and microbial communities. The gut microbiota has been widely recognized as a key factor in metabolic diseases (5). Meanwhile, as the second largest microbial niche after the gut, the oral microbiome has gained attention for its potential roles in disease. Notably, tongue diagnosis, a key component of traditional Chinese medicine, has long been used in the clinical assessment of GLMD (4). Modern studies further show that tongue-coating phenotypes are closely associated with the oral microbial community (6), indicating that they may serve as an observational window into GLMD state.

Existing oral-systemic microbiome reviews have advanced the understanding of oral microbial ecology, oral-gut microbial transmission, and associations between oral dysbiosis and systemic diseases (7, 8). However, many available discussions either address oral-systemic links broadly or focus on individual diseases. In contrast, this review uses the established GLMD disease concept as an integrative lens to organize current evidence across three levels: disease-associated oral microbial signatures, plausible mechanistic links with host metabolic and inflammatory pathways, and translational opportunities for non-invasive risk assessment and individualized intervention. This approach does not assume that oral dysbiosis is a common primary cause of all GLMD-related conditions; rather, it asks whether scattered findings from individual metabolic diseases can be interpreted as recurrent microbial patterns, shared functional modules, and disease-specific oral microbial signatures within the GLMD context.

By adopting this framework, we aim to clarify where current evidence supports common oral microbiome-related patterns across GLMD, where disease-specific differences and inconsistent findings remain important, and where causal inference and clinical translation remain limited.

## 2 GLMD as a disease concept for integrating oral microbiome evidence

The concept of glucolipid metabolic disorders (GLMD) has been proposed as an integrative framework for interrelated disorders of glucose and lipid metabolism. Previous work on GLMD emphasized that dyslipidemia, type 2 diabetes mellitus, nonalcoholic fatty liver disease, and related cardiometabolic complications frequently coexist in clinical practice and share closely connected pathological backgrounds, including neuroendocrine-immune dysregulation, insulin resistance, oxidative stress, inflammation, and intestinal microbiota imbalance (9). In China, a 2019 expert consensus and a 2021 clinical practice guideline further recognized GLMD, also described in traditional Chinese medicine as “Danzhuo,” as a clinically meaningful category for integrated diagnosis, prevention, and management (4, 10). Some papers have supported this integrated view. Liver-adipose tissue crosstalk, partly mediated by the FGF21-adiponectin axis, has been proposed as an important mechanism coordinating glucose and lipid homeostasis in GLMD (11). The hypothalamus-pituitary-adrenal axis, as a central component of the neuroendocrine-immune network, has also been implicated in systemic metabolic regulation in GLMD (12). In addition, metaflammation provides a shared pathological context, as inflammatory signaling pathways such as NLRP3/caspase-1/IL-1, NF-κB, p38 MAPK, IL-6/STAT3, and PI3K/AKT participate in metabolic dysregulation and cardiometabolic comorbidity (13).

Building on this established disease concept, the present review asks whether oral microbiome findings across GLMD-related conditions can also be organized into shared and disease-linked patterns. Diabetes, obesity, dyslipidemia, MASLD/NAFLD, hypertension, and cardiovascular disease are not isolated clinical entities. Obesity and insulin resistance can promote hyperglycemia, dyslipidemia, hepatic steatosis, and blood pressure elevation, whereas chronic low-grade inflammation, oxidative stress, endothelial dysfunction, and lipid abnormalities contribute to atherosclerosis and cardiovascular complications. Similarly, gut microbial dysbiosis has been widely recognized as a common biological background in metabolic diseases (5). These clinical and mechanistic connections provide a rationale for examining whether oral microbial alterations reported in individual metabolic diseases may also contain recurrent signals relevant to GLMD as a whole.

Current oral microbiome evidence remains scattered, but it is not entirely disease-specific or random. Several taxa or microbial groups, including *Streptococcus, Prevotella, Porphyromonas gingivalis, Haemophilus, Tannerella forsythia, Fusobacterium, Aggregatibacter, Rothia*, and *Veillonella*, have been reported across different GLMD-related conditions, although their abundance, direction of change, and clinical relevance vary according to disease phenotype, sampling site, periodontal status, population characteristics, and analytical approach. A recent scoping review of oral microbiome studies in diabetes, arterial hypertension, and obesity found partially overlapping microbial patterns across metabolic diseases, while also highlighting substantial heterogeneity and the absence of a universal oral microbial signature (14). Therefore, this review does not assume that a single common oral microbiome-mediated axis has already been established for all GLMD-related diseases. Instead, it uses the GLMD disease concept to evaluate whether dispersed findings can be linked through recurrent microbial patterns, shared functional modules, and biologically plausible mechanisms, including periodontal inflammation, nitrate-nitrite-nitric oxide metabolism, oral-gut microbial transmission, hematogenous dissemination, and microbial metabolites affecting inflammation, oxidative stress, glucose metabolism, lipid metabolism, and barrier function.

## 3 Oral microbiome

### 3.1 Formation and composition of the oral microbiome

From the inner to the outer rings, the hierarchy represents the levels of phylum, class, order, family, and genus. Different colors denote distinct taxa, and the size of each sector indicates the number of species contained within each taxonomic level.

**FIGURE 1:**
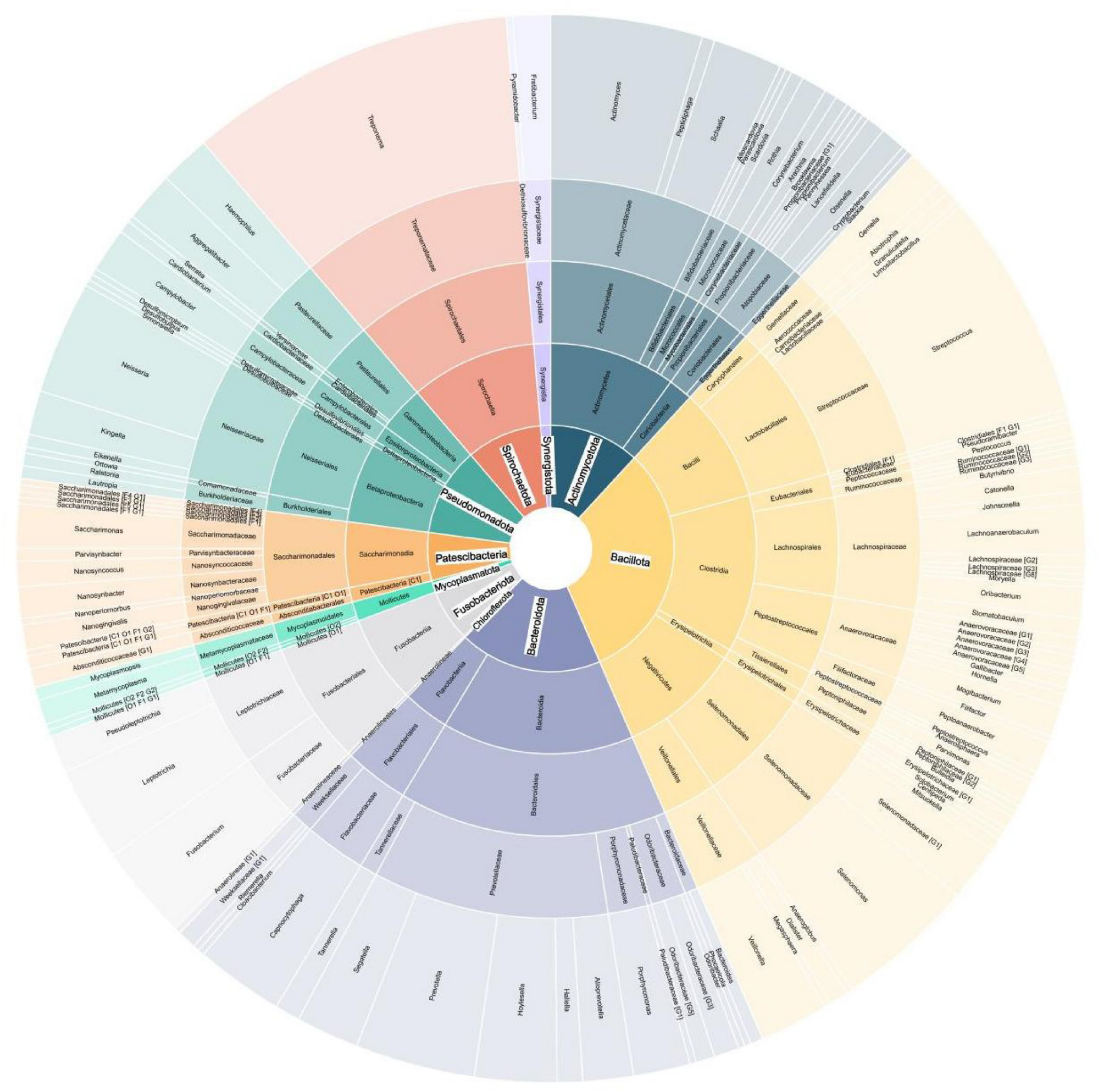
Circular visualization of the taxonomic composition of the oral microbiota.

More than three centuries ago, Antony van Leeuwenhoek first observed the microbes in his mouth with a self-made microscope (15), establishing the origins of microbiology and marking the discovery of the oral microbiota. To date, over 700 microbial species have been identified in the human oral cavity, primarily belonging to several dozen genera across seven major phyla (16). The primary taxa of the oral cavity in healthy individuals consist of the genera (as shown in Figure 1), including *Streptococcus*, *Granulicatella*, *Veillonella*, *Gemella* (*Bacillota*), *Neisseria*, *Haemophilus* (*Pseudomonadota*), *Corynebacterium*, *Rothia*, *Actinomyces* (*Actinomycetota*), *Prevotella*, *Capnocytophaga*, *Porphyromonas* (*Bacteroidota*), and *Fusobacterium* (*Fusobacteriota*), present in different proportions in the diverse locations of the oral cavity. Although present in relatively low abundance, archaea, microeukaryotes (including fungi, amoebae, and flagellates), and viruses are also part of the oral microbiome. Their distinct metabolic traits suggest that they may contribute to both ecological interactions and pathogenic processes within the oral cavity. Common fungal genera include *Candida*, *Malassezia*, *Cladosporium*, *Saccharomyces*, and *Penicillium*, and are widely distributed in the oral cavity. In contrast, archaea (*Methanobrevibacter* spp.), amoebae (*Entamoeba gingivalis*), and flagellates (*Trichomonas tenax*) primarily live in the periodontal pocket.

The Human Oral Microbiome Database (HOMD)^1^ was established in 2007 and is the first website to provide tools for systematically describing human oral microorganisms. It offers comprehensive information on bacteria in the human mouth and aerodigestive tract, including the pharynx, nasal passages, sinuses, and esophagus. Of the oral taxa, 49% are named, 21% unnamed but cultivated, and 29% are known only as uncultivated phylotypes (17). The diversity of the oral microbiota reflects the anatomical complexity of the mouth, and different oral sites support distinct microbial communities. The oral cavity consists of various habitats, such as the hard surface of the tooth enamel (both above and below the gumline), the keratinized surfaces of the palate, gingiva, tongue papillae, and the soft surfaces, such as the buccal mucosa, which provide different environments in which microorganisms can flourish. Accordingly, microbial profiles differ markedly among dental plaque, the tongue dorsum, and keratinized gingiva (7). The oral cavity, as the initial site of the digestive system, maintains a relatively stable temperature, and saliva exhibits a pH range of 6.5–7. Together, these conditions shape an environment that favors the establishment and maintenance of the oral microbiota.

The oral cavity of neonates is generally considered sterile before birth, with the establishment of the oral microbiome occurring postnatally. Maternal transmission, feeding practices, and close contact with caregivers mainly shape early microorganism colonization. As age increases, the oral microbiome gradually stabilizes (18). However, its composition continues to evolve throughout life. The study suggests that oral microbial communities tend to converge toward a more uniform profile in midlife, whereas in older adults, they become more diverse, with increased representation of typically low-abundance taxa (19). Throughout the lifespan, a variety of influences continually shape the oral microbiota, including diet, stress, oral hygiene practices, host genetics, systemic diseases, medication use (particularly antibiotics), alcohol consumption, and smoking (20). Among these, chronic diseases have been identified as a primary determinant of the oral microbiome’s composition (19). These influences underscore that the oral microbiome changes over time rather than remaining constant.

Among human body sites, the oral cavity is often reported to host a relatively stable microbial community with comparatively high alpha diversity (21). In a large-scale analysis that produced a biogeographical map of the human microbiota, researchers investigated temporal dynamics across body habitats. The analysis indicated comparatively high temporal stability in several oral habitats, particularly the tongue dorsum. The microbial community on the tongue dorsum showed good temporal stability (21). The stability of the oral ecological niche was further confirmed. A study by Nearing et al. examined the influence of lifestyle, anthropometrics, and diet on the oral microbiome (22). They found that although numerous factors were significantly associated with its composition, no single factor accounted for more than 2% of the microbial variance. Each of these factors was associated with only minor shifts in the overall taxonomic composition of the oral microbiome, suggesting that future biomarker identification for several diseases related to the oral microbiome can be undertaken although confounding and site-specific variability must still be carefully controlled. These findings support the potential of the oral microbiome as a biomarker source, although its clinical use still requires standardized sampling, longitudinal validation, and careful control of oral-health-related confounders.

### 3.2 Tongue coating formation and oral microbiota

Tongue coating is a complex, multilayered biofilm that covers the dorsal surface of the tongue. Under normal physiological conditions, it appears as a thin, white, and moist layer. The tongue coating forms by exfoliating epithelial cells, saliva, resident microbes, food debris, and leukocytes. Together, these components create a microenvironment that favors microbial growth (23). The specialized structure of the tongue’s dorsal mucosa, characterized by numerous fissures and folds, combined with the oxidation and decomposition of food residues, as well as the tongue’s warm and humid surface, creates a mildly oxygen-limited environment that facilitates microbial colonization. Each epithelial cell on the dorsal tongue surface can carry around 100 bacteria. This density is substantially higher than at many other oral sites, making the tongue a microbially rich niche. Oral epithelial cells renew approximately every 2.7 h, leading to rapid biofilm shedding, which can affect the stability of experimental results. By comparison, biofilms on the tongue shed more slowly. Epithelial turnover from the basal layer to the surface typically takes 3–7 days, thereby providing greater microbial stability. This cycle offers greater stability, further enhanced by synergistic interactions, signaling pathways, and antagonistic effects between epithelial cells and microbes (24, 25). For these reasons, the tongue is often selected as a sampling site in oral microbiome studies.

Although factors such as individual physiological conditions, dietary habits, and regional environments contribute to heterogeneity in tongue coating microbiota, some studies report that disease-related microbial patterns in tongue coatings remain detectable even after accounting for lifestyle factors, suggesting their potential as non-invasive biomarkers (26). Healthy oral microbiota may harbor a “core microbiome” (27), while different diseases often exhibit disease-specific microbial profiles. In traditional Chinese medicine, tongue coating is considered a reflection of overall health, with variations in color, thickness, texture, and moisture indicating different pathological states. Examples include fissured tongue (associated with hyposalivation, candidiasis, diabetes mellitus, vitamin B deficiency, lichenoid reactions, and Sjögren syndrome) and depapillated tongue (indicative of nutritional deficiencies, xerostomia, local trauma, or candidiasis) (28). Recent microbiome studies show variations in microbial abundance across different tongue coating morphologies (6). For instance, in patients with type 2 diabetes mellitus (T2DM), yellow tongue coating is associated with higher levels of *Lactobacillus* spp. compared to non-yellow coatings (29). In nonalcoholic fatty liver disease (NAFLD) patients, the tongue microbiota differs by coating color: *Corynebacterium, Moraxella, Ottowia, Lactobacillus, Johnsonella, Tissierella*, and *Enterobacter* were found only in the White Coating Group, while *Shuttleworthia, Simonsiella, Desulfobulbus*, and *Mycoplasma* were unique to the yellow coating group. In terms of relative abundance, *Neisseria* was dominant in the white coating group, while *Prevotella* was dominant in the yellow coating group (30). Another study on chronic gastritis patients indicated that the microbial changes in the oral cavity may be associated with the formation of greasy tongue coatings (31).

### 3.3 Oral-gut microbiome axis

As the gateway to the digestive system, the oral cavity is one of the earliest sites of microbial colonization. Its microbial community can influence downstream sites, including the respiratory and gastrointestinal tracts. Building on these observations, researchers have proposed several cross-organ microbial axes, including the oral–gut–liver, oral–lung, and oral–gut–brain axis. Among them, the oral-gut axis is an essential pathway. Further down the digestive tract, the gut contains a highly diverse microbial community shaped by factors such as genetics, diet, lifestyle, and environmental exposures. Despite their anatomical continuity, the microbial communities of the mouth and gut differ markedly. This separation is maintained by the oral–gut barrier, including gastric acid, bile, pancreatic enzymes, and intestinal colonization resistance (32).

**FIGURE 2:**
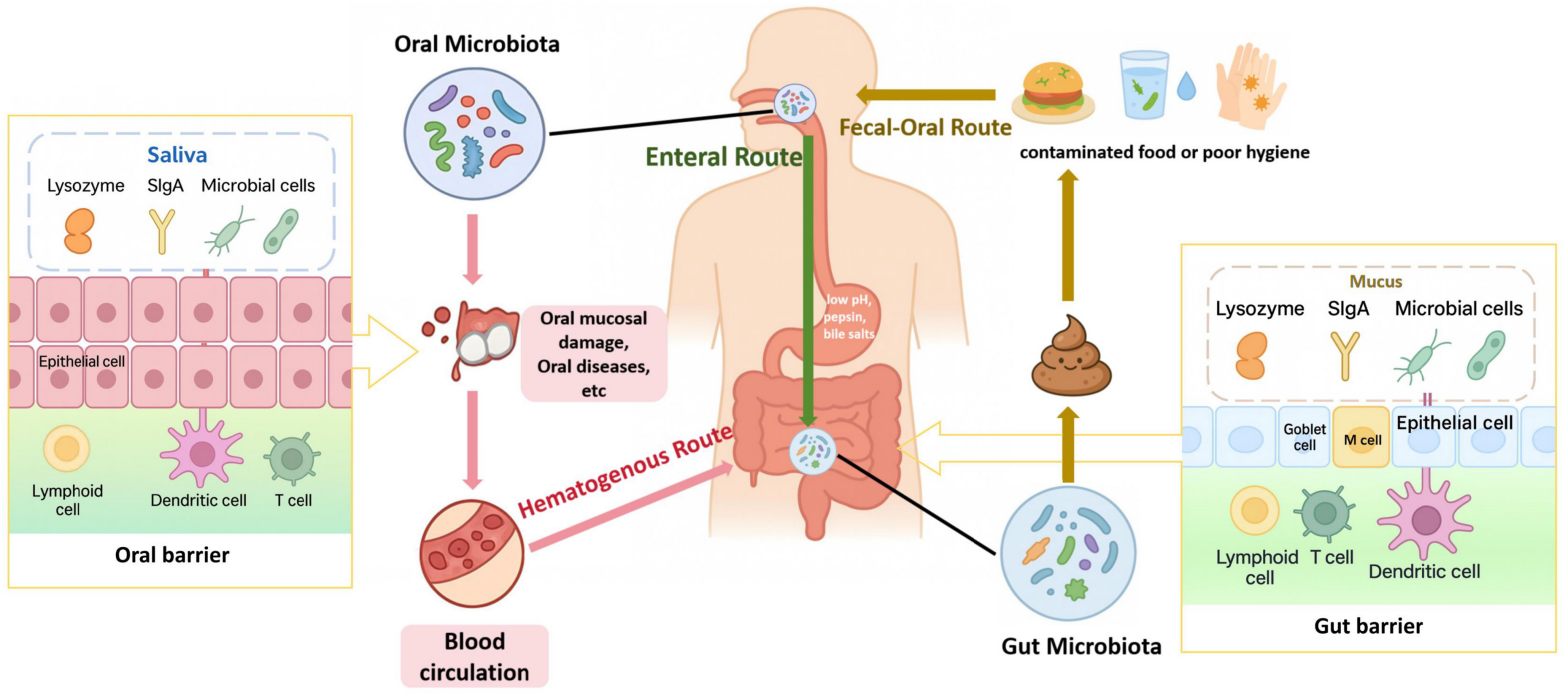
Microbial translocation pathways in the oral-gut axis.

Oral microorganisms can translocate to the gut and other distant organs through two primary routes (Figure 2): the digestive route, in which oral bacteria pass through the gastrointestinal tract to colonize the intestine, and the hematogenous route, in which bacteria enter the bloodstream during oral disease or tissue damage and spread systemically. Conversely, microbes can move from the gut to the mouth through fecal–oral transmission, such as contact with contaminated hands, food, or water. The oral–gut connection also involves microbial metabolites that act as signaling molecules. Metabolites such as short-chain fatty acids (SCFAs), trimethylamine N-oxide (TMAO), indoles and their derivatives, bile acids, and lipopolysaccharides (LPS), can circulate systemically and affect inflammation, oxidative stress, metabolic pathways, and barrier function. These actions may provide biologically plausible links with metabolic disorders (8).

The schematic outlines two major pathways linking the oral and gut microbiomes: a hematogenous route, in which oral microbes enter the bloodstream, and an enteral route through the gastrointestinal lumen. Additional fecal–oral transmission can occur via contaminated food or poor hygiene. Several additional defenses act within the gastrointestinal tract. Gastric acid (pH ∼2) can disrupt bacterial cell walls and membrane proton gradients. Pepsin further degrades proteins, and bile salts damage membranes, denature proteins, and bind essential ions.

The left panel highlights the oral barrier, where stratified squamous epithelium and saliva act as primary defenses. Saliva contains lysozyme, secretory immunoglobulin A (SIg A), and other antimicrobial proteins. Immune cells such as lymphoid cells, T cells, and dendritic cells, also help maintain immune balance and limit microbial passage. The right panel shows the gut barrier, which is organized as a single epithelial cell layer covered by mucus secreted by goblet cells. Specialized M cells take up luminal antigens and pass them to immune cells beneath the epithelium.

Early hypotheses suggested that oral microbes could colonize the gut primarily when intestinal dysbiosis was present. However, a 2019 study demonstrated that the transmission and subsequent colonization of oral bacteria in the gut are also common among healthy individuals. Although the extent varies among individuals, many oral species can be transferred to the gut (33). In that study, roughly one-third of identifiable salivary microbes were also detected as colonizers in the gut, accounting for at least 2% of the classifiable microbial abundance in fecal samples. The estimates were considered conservative due to stringent thresholds and detection limits inherent to metagenomic sequencing. The authors suggested that most oral species can reach the gut, implying that the oral cavity may serve as an endogenous reservoir of intestinal microbial strains (33). Consistent with this, a report from Segata et al. in Genome Biology found that oral cavity and stool bacteria overlapped in nearly half (45%) of subjects in the Human Microbiome Project (34). One analysis reported that oral microbial composition could be inferred from fecal microbiota patterns (35). Several bacterial taxa are frequently transmitted from the oral cavity to the gut, forming coherent strain populations along the gastrointestinal tract (36). In addition to this natural translocation, certain conditions weaken the oral–gut barrier and make colonization by oral taxa easier. For instance, insufficient gastric acid secretion (hypochlorhydria), particularly with the use of proton pump inhibitors (PPIs), can shift gut microbial communities toward a profile resembling that of the mouth. Antibiotics can also weaken native gut populations, reducing colonization resistance and creating a niche for oral bacteria. Moreover, specific oral species, such as the notoriously acid-tolerant *Streptococcus mutans*, can withstand gastrointestinal conditions and colonize the gut (8).

The oral–gut microbiome axis is closely associated with the onset and progression of metabolic diseases. Recent studies have identified connections between NAFLD and dysbiosis of both the oral and gut microbiota. Notably, *Porphyromonas gingivalis* (*P. gingivalis*) has been reported to disrupt gut microbial balance and exacerbate NAFLD by disrupting metabolic and immunologic homeostasis (37). Using metabolic labeling, researchers demonstrated experimentally that viable *P. gingivalis* can migrate to the gastrointestinal tract and induce insulin resistance-like metabolic changes (38). Li et al. uncovered its role in exacerbating myocardial ischemia reperfusion injury (MIRI), suggesting that elevated levels of oral *Fusobacterium nucleatum* and gut *Lactobacillus* may play intermediary roles in this pathological process (39). A study by Chen et al. demonstrated microbial transmission from the oral cavity to the gut in hypertensive patients, identifying 16 species across five genera as “oral-gut transmitters.” Among these, species of the genus *Veillonella* were the most consistently enriched and prevalent transmitters (40). Collectively, these findings support the possibility that the oral-gut axis participates in host metabolic and immunometabolic processes.

Although identical species may be found in both sites, this pattern does not always imply ongoing microbial transfer. Based on current clinical observations and experimental findings, the oral–gut microbiome axis and its associated transmission and effects appear to be bidirectional. Although a strong correlation has been established, the specific mechanisms and causal links are still uncertain[8]. Given the remarkable microbial diversity and the intricate interactions between oral and gut microorganisms, as well as with the host, both communities influence health and can contribute to disease development. Compared with the gut microbiota, the oral microbiome offers a more convenient approach, and oral sampling is generally more acceptable to participants and more suitable for repeated collection than fecal sampling, which makes the oral microbiome a promising candidate for noninvasive biomarker development, provided that site-specific sampling effects, temporal variability, and reproducibility across cohorts are adequately addressed.

## 4 The oral microbiome in GLMD

GLMD provides a framework for considering metabolic disorders in a broader clinical context, highlighting how metabolic conditions frequently co-occur and interact. The concept extends beyond single metabolic diagnoses by integrating shared abnormalities and the comorbid patterns that often accompany them.

**FIGURE 3:**
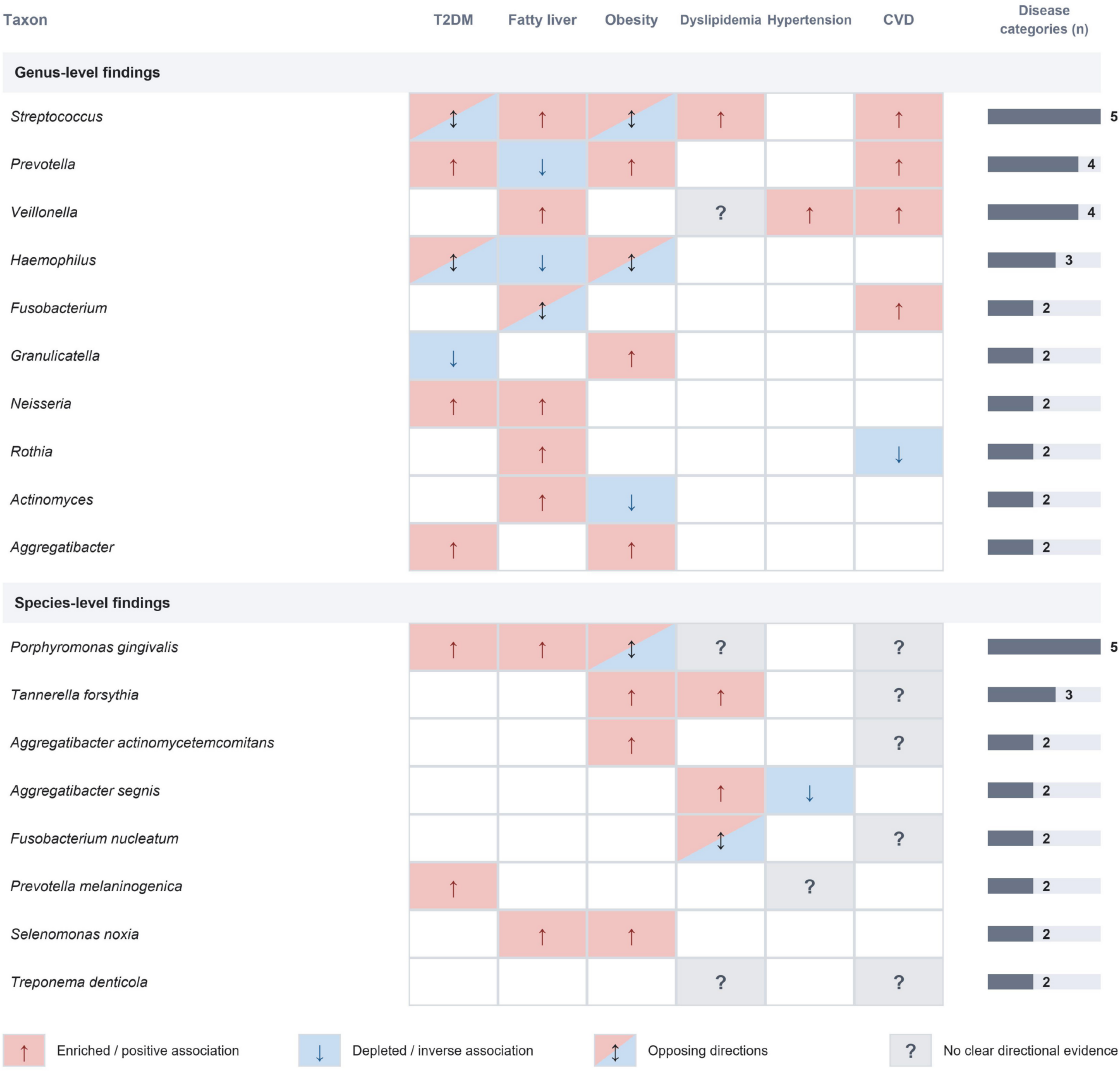
Cross-disease recurrence and direction of reported oral microbial associations in GLMD.

The relationship between the human microbiome and metabolic diseases is well recognized, and the oral microbiome interacts with host physiology in ways that may be relevant to the onset and progression of GLMD. Across various metabolic diseases, several key microbial taxa have been repeatedly detected (see Figure 3), including *Streptococcus*, *Prevotella*, *P. gingivalis*, *Haemophilus*, *Tannerella forsythia*, and *Veillonella*. These taxa appear repeatedly across studies, suggesting that certain oral microbes may have consistent associations with systemic metabolic traits, and they may form a core oral microbiota–systemic metabolism network.

Comparisons across studies show that several oral taxa associated with cardiovascular disease are also observed in other metabolic disorders. This overlap may provide context for the increased cardiovascular risk observed in individuals with multiple metabolic comorbidities. These patterns suggest that changes in the oral microbiota may be relevant across metabolic conditions. As summarized in Table 1, substantial heterogeneity in study design, population characteristics, oral sampling, and analytical methods complicates the interpretation and cross-study comparison of associations between the oral microbiota and metabolic diseases.

**TABLE 1:** Characteristics and principal findings of included oral-microbiome studies.

Study	Disease/source	Design, population and sample	Oral sample	Microbiome method	Main taxa findings	Quantitative results	Confounders/ adjustment
Kozarov et al. (41)	Cardiovascular disease/carotid atherosclerosis Original study	Single-patient proof-of-concept tissue study One 74-year-old male carotid endarterectomy patient with dentures and 80% carotid occlusion Total: 1 patient Groups: No comparison group	No oral sample collected	Cell invasion assay with immunofluorescence/ deconvolution microscopy; species-specific SYBR Q-PCR targeting 16S rRNA genes	*Porphyromonas gingivalis* and *Actinobacillus* (*Aggregatibacter*) actinomycetemcomitans were detected in the carotid plaque; this study did not estimate an increase relative to a control group.	Q-PCR: *P. gingivalis* log 4.273 and *A. actinomycetem*comitans log 4.779 in the analyzed DNA sample; extrapolated to approximately 1.9 × 10^5^ and 6.0 × 10^5^ organisms, respectively, in the resected tissue.	No confounder adjustment; single-patient study. Plaque was washed after resection, and peripheral-blood leukocyte DNA was used as a negative specificity control.
Goodson et al. (42)	Overweight/obesity Original study	Cross-sectional comparison using a convenience control population Boston participants; overweight group comprised Caucasian women aged 20–45 years with BMI 27–32 and in good general health; the controls were healthy adults from periodontal studies Total: 545 participants Groups: 313 overweight women; 232 healthy controls	1–3 mL whole unstimulated saliva collected by expectoration	Checkerboard whole-genomic DNA probe analysis of 40 oral bacterial species	Seven of 40 species had a median percentage difference > 2% in overweight women. *Selenomonas noxia* was higher and was the principal classifier; the text also reports higher median percentages for most measured *Firmicutes* and significant increases among species from other phyla.	*S. noxia* > 1.05% of measured salivary bacteria classified overweight status with 98.4% sensitivity and 80.2% specificity. Mann-Whitney U tests were adjusted for multiple comparisons.	No multivariable adjustment. Eligibility restricted medication and systemic illness; controls had periodontal health and no recent systemic antibiotics. Smoking was not excluded. Authors identify use of a convenience control population rather than selected cohorts as a major limitation.
Koren et al. (43)	Cardiovascular disease/carotid atherosclerosis Original study	Matched case-control, multi-site microbiome study Patients undergoing carotid endarterectomy after minor ischemic stroke, transient ischemic attack, or amaurosis fugax; population-based controls matched by sex and without current or previous cerebrovascular disease Total: 30 participants Groups: 15 atherosclerosis patients; 15 age- and sex-matched healthy controls	Mouth swab collected by a nurse	V1-V2 16S rRNA gene barcoded 454 pyrosequencing (27F/338R), QIIME analysis with 97% OTUs; qPCR quantified bacterial DNA in plaque	No species-level oral OTU discriminated cases from controls. In plaque, *Chryseomonas* was detected in all samples and *Veillonella* and *Streptococcus* in most. Oral *Fusobacterium* abundance correlated positively with total and LDL cholesterol; oral *Streptococcus* correlated positively with HDL and ApoAI, whereas *Neisseria* correlated negatively with HDL and ApoAI. These are biomarker correlations, not case-control increases.	Plaque bacterial DNA correlated with plaque leukocytes (Spearman rho = 0.68, *P* = 0.009). Oral *Fusobacterium* vs. cholesterol and LDL: rho = 0.63 (*P* = 0.028) and 0.75 *(P* = 0.005). *Streptococcus* vs. HDL and ApoAI: rho = 0.88 (*P* = 0.0001) and 0.70 (*P* = 0.01). Neisseria vs. HDL and ApoAI: rho = -0.65 (*P* = 0.02) and -0.74 (*P* = 0.005). Combined *Veillonella* + *Streptococcus* abundances in mouth and plaque: rho = 0.60, *P* = 0.03.	Controls were age- and sex-matched; patients completed questionnaires on diseases, lifestyle, and medication. Reported microbiome-biomarker analyses were correlations without a stated multivariable confounder-adjusted model.
Hyvärinen et al. (44)	Cardiovascular disease/coronary artery disease Original study	Cross-sectional angiography-based clinical study Native Finnish symptomatic patients undergoing diagnostic coronary angiography; mean age 63 ± 9 years, 65% male Total: 492 participants Groups: No significant CAD *n* = 119; stable CAD *n* = 179; acute coronary syndrome (ACS) *n* = 166; ACS-like symptoms without significant CAD *n* = 28	At least 2 mL paraffin-stimulated whole saliva collected by expectoration	Species-specific TaqMan quantitative real-time PCR for A. actinomycetemcomitans, P. gingivalis, P. intermedia and T. forsythia; results in genomic equivalents/mL saliva	Among pathogen-positive participants, salivary *A. actinomycetemcomitans* levels were higher in stable CAD and ACS than in the no-significant-CAD group. Other measured pathogens did not differ significantly among angiographic groups.	Median *A. actinomycetemcomitan*s levels were 6-fold higher in stable CAD (*P* = 0.014), 2.6-fold higher in ACS (*P* = 0.044), and 4,550-fold higher in the small ACS-like/no-CAD group (*P* = 0.023) versus no significant CAD. Per 10-fold salivary increase, adjusted OR = 7.47 (95% CI 1.57–35.5; *P* = 0.012) for stable CAD and OR = 4.31 (95% CI 1.06–17.5; *P* = 0.041) for ACS.	Multivariable logistic regression adjusted for age, BMI, sex, hypertension, dyslipidemia, diabetes and smoking; interaction terms with age, sex, smoking, diabetes, hypertension and BMI were tested. The ACS-like/no-CAD group was excluded from regression because *n* = 28.
Wu et al. (45)	Obesity Original study	Age- and sex-matched cross-sectional case-control study Chinese adults aged 20–40 years with good systemic and periodontal health; obesity BMI ≥ 30 and normal weight BMI 18.5–20 Total: 62 participants Groups: Obesity *n* = 33; normal weight *n* = 29 (80 screened; 18 excluded for periodontitis or < 20 teeth)	5 mL spontaneous whole unstimulated saliva, morning collection after ≥ 8 h without food or drink	V3-V4 16S rRNA gene amplicon sequencing (338F/806R), Illumina MiSeq PE300; QIIME-based analysis	Obesity: increased *Prevotella*, *Granulicatella*, *Peptostreptococcus, Solobacterium, Catonella* and *Mogibacterium*; decreased *Haemophilus*, *Corynebacterium, Capnocytophaga* and *Staphylococcu*s. Alpha diversity and richness were lower in obesity.	Differential abundance and diversity were reported as statistically significant in the original analyses; the main text excerpt does not provide a single effect size suitable for all listed genera.	Age- and sex-matched. Strict exclusions included systemic disease, medications, smoking, pregnancy/lactation, menopause, recent antibiotics or hormonal contraceptives, < 20 teeth and periodontal attachment loss. No multivariable confounder-adjusted association model was reported.
Burleigh et al. (46)	Cardiovascular physiology/nitrate-nitrite pathway Original study	Single-arm acute dietary nitrate challenge with cross-sectional microbiome association analysis 25 healthy adults (age 27 ± 7 years; 11 female) with good cardiovascular and oral health and no prescribed medication Total: 25 enrolled; 24 included in sequencing analyses Groups: Low nitrate-reducer abundance ( < 50%) *n* = 7; high abundance ( > 50%) *n* = 17 after exclusion of one sample with < 5000 reads	Posterior dorsal tongue scrape for microbiome; serial unstimulated saliva collected with an under-tongue oral swab	V3-V4 16S rRNA amplicon sequencing (341F/806R), Illumina MiSeq; QIIME 1.8, 97% OTUs	Seven known nitrate-reducing species were identified; *Prevotella* melaninogenica and *Veillonella* dispar were most prevalent. This was not a disease case-control study. Participants with a higher summed abundance of nitrate reducers generated salivary nitrite more rapidly; the low-abundance group had higher alpha diversity.	Nitrate-reducer abundance correlated with peak salivary nitrite change (*r* = 0.44, *P* = 0.03) and salivary nitrite AUC (*r* = 0.40, *P* = 0.05). Low vs. high groups: 40.99 ± 6.11% vs. 62.64 ± 6.92% nitrate-reducing OTUs; time to peak salivary nitrite 3.0 ± 0.6 vs. 1.6 ± 1.0 h (*P* = 0.04). Low group had 1279 ± 136 vs. 1098 ± 129 OTUs (*P* < 0.001) and Shannon index 5.9 vs. 4.9 ± 0.6 (*P* = 0.002).	Diet was recorded for 7 days; exercise, caffeine and oral-hygiene behaviors were standardized before testing. Groups did not differ in nitrate-containing food intake or baseline physiological variables. Analyses included repeated-measures/mixed ANOVA and Spearman correlations, but no multivariable confounder adjustment.
Wang et al. (47)	Hyperglycemia/type 2 diabetes Original study	Cross-sectional community-based study Elderly Chinese residents from Shanghai community health service centers Total: 150 participants Groups: Normal fasting glucose < 6.1 mmol/L *n* = 76; high 6.1–7 mmol/L *n* = 23; very high > 7 mmol/L *n* = 51	Saliva collected after 12 h fasting and without tooth brushing, food intake or smoking	V3-V4 16S rRNA amplicon sequencing (338F/806R), Illumina MiSeq; QIIME 1.8, 97% OTUs; LEfSe	Very-high-glucose group: enrichment of *Leptotrichia, Bulleidia, Staphylococcus* and *Catonella* (genus level; LEfSe LDA ≥ 2). *Leptotrichia* was higher versus the high-glucose group and showed a nonsignificant increasing trend versus normal.	*Leptotrichia*: VH vs. H *P* = 0.014; VH vs. normal trend *P* = 0.085. Per sample 38,706 ± 4,321 high-quality reads; 7,263 OTUs at 97% identity. LEfSe features used LDA score ≥ 2.0.	No differences in age, sex, smoking or drinking status across groups, but body weight/BMI and metabolic measures differed. Excluded serious disease and antibiotic use in the previous 3 months. Analyses were unadjusted group comparisons/LEfSe rather than multivariable adjustment.
Kamińska et al. (48)	Dyslipidemia treatment context/periodontal biofilm Original study	*In vitro* experimental study Planktonic monocultures and a five-species oral biofilm model; no human or animal participants Total: Not applicable (laboratory replicates) Groups: Untreated controls versus atorvastatin, fluvastatin, lovastatin and simvastatin conditions in developing and established biofilms	No clinical oral sample	Anaerobic planktonic growth/MIC assays; multispecies biofilm assay; species-specific quantitative PCR and scanning electron microscopy	All four statins inhibited *P. gingivalis* and reduced cumulative load in developing and established biofilms; simvastatin had the strongest selective effect. This is a treatment effect, not evidence that *P. gingivalis* is increased in dyslipidemia.	Simvastatin reduced *P. gingivalis* counts by > 1,300-fold relative to untreated control.	Not applicable to an *in vitro* experiment; untreated and vehicle controls were used.
Chen et al. (49)	Type 2 diabetes mellitus Original study	Cross-sectional case-control study Chinese adults: hospitalized T2DM patients and healthy examination controls Total: 442 participants Groups: T2DM *n* = 280 (160 male/120 female); controls *n* = 162 (100 male/62 female)	Buccal mucosa swab; no eating, tooth brushing or flossing for 2 h before collection	V1-V2 16S rRNA amplicon sequencing (27F/355R), Illumina HiSeq 2500; QIIME 1.9.1, LEfSe and PICRUSt	T2DM: increased *Firmicutes/Bacteroidete*s ratio and enrichment of *Neisseria, Streptococcus, Haemophilus* and *Pseudomonas*; *Acinetobacter* was enriched in controls. T2DM had higher richness and Shannon diversity.	F/B ratio 7.597 ± 6.737 vs. 2.743 ± 4.024 (*P* < 0.0001). Chao1 3300 ± 446.7 vs. 3079 ± 516.8 (*P* = 0.0011); Shannon 5.777 ± 1.002 vs. 4.829 ± 0.9578 (*P* < 0.0001). Community separation: ANOSIM *r* = 0.9796 unweighted and *r* = 0.2448 weighted UniFrac (both *P* = 0.0001).	Exclusions addressed systemic disease, recent antibiotics/steroids/probiotics, dietary extremes and active oral disease. Authors state matching on age, sex and BMI, but the reported table shows age 48 ± 8 vs. 46 ± 6 (*P* < 0.001) and BMI 27.1 ± 0.8 vs. 20.1 ± 1.2 (*P* < 0.001); no multivariable adjustment was reported.
LaMonte et al. (50)	Hypertension Original study	Prospective cohort with baseline cross-sectional analysis Community-dwelling postmenopausal women aged 53–81 years in the Buffalo OsteoPerio/Women’s Health Initiative cohort Total: 1215 women at baseline Groups: Normotensive *n* = 429; untreated elevated BP *n* = 306; prevalent treated hypertension *n* = 480. Prospective risk set *n* = 735, with 375 incident cases over mean 10.4 years.	Subgingival plaque collected at standardized sites using fine paper points inserted into the gingival sulcus for 10 s	V3-V4 16S rRNA amplicon sequencing, Illumina MiSeq 2 × 300 bp; approximate species assignment at 97% similarity against HOMD	Higher incident-hypertension risk: *S. anginosus*, *S. salivarius, Fretibacterium* sp. oral taxon 362, *S. infelix, Prevotella* sp. oral taxa 526/292/376, *Megasphaera* sp. oral taxon 123, *Capnocytophaga sp*. oral taxon 903 and *S. lactarius*. Lower risk: *N. subflava, Bergeyella* sp. oral taxon 907, *G. morbillorum, Leptotrichia* sp. oral taxon 212 and *A. segnis.*	Age-adjusted HRs per SD abundance were 1.10–1.16 for 10 positive associations and 0.82–0.91 for 5 inverse associations. Thirteen remained nominally significant after full adjustment, but none remained significant after multiple-comparison correction.	Cox models progressively adjusted for demographic/clinical variables, smoking, BMI, baseline BP, treated diabetes, diet quality, physical activity and statin use; stratified analyses examined age, smoking, BMI and BP categories.
Prince et al. (51)	Metabolic syndrome Original study	Case-control study nested in a South African cohort Western Cape, South African adults classified by 2009 Joint Interim Statement metabolic-syndrome criteria Total: 128 participants Groups: Metabolic syndrome *n* = 62; without metabolic syndrome *n* = 66	Four subgingival plaque samples collected by wood toothpick from bilateral premolar regions after 12 h fasting and without brushing, food or smoking	Ion 16S Metagenomic Kit covering V2/V4/V8 and V3/V6-7/V9 regions; Ion S5 GeneStudio sequencing; QIIME 2, 97% OTUs	Higher in MetS: *A. dentalis, A. naeslundii, A. viscosus, C. matruchotii, L. buccalis* and *S. sanguinis*. Lower in MetS: *A. odontolyticus, C. gracilis, F. canifelinum, F. nucleatum, F. periodonticum, H. parainfluenzae* and *V. rogosa*e. Thus, the pre-existing map’s uniformly “increased” directions were not supported by the source table.	Alpha richness: 275 vs. 253 taxa; beta diversity indicated 24% dissimilarity. Examples of relative abundance (without MetS vs. MetS): *A. naeslundii* 1.48 vs. 4.03 (*P* < 0.001), C. matruchotii 2.97 vs. 7.91 (*P* < 0.001), *F. nucleatum* 7.09 vs. 4.62 (*P* < 0.001), H. parainfluenzae 10.10 vs. 3.94 (*P* < 0.001), *S. sanguinis* 0.43 vs. 1.56 (*P* < 0.001). In multivariable logistic regression, *C. gracilis* remained significant (OR 0.29, 95% CI 0.12–0.68; *P* = 0.005).	Multivariable logistic regression assessed MetS, periodontitis and gingival bleeding; metabolic-factor correlations were also examined. Groups differed in cardiometabolic characteristics by definition; sex distribution also differed.
Li et al. (52)	Type 2 diabetes mellitus Original study	Cross-sectional case-control multi-omics study Adults with T2DM and orally healthy controls examined at Xijing Hospital Total: 20 participants Groups: T2DM *n* = 10; healthy controls *n* = 10; each participant contributed saliva and supragingival plaque	≥ 10 mL unstimulated whole saliva by expectoration; supragingival plaque from cervical premolar/molar surfaces using sterile Gracey curettes, collected before breakfast after 12 h without oral hygiene, food, drink or gum	Shotgun metagenomic sequencing on MGISEQ-2000; untargeted LC-MS metabolomics	T2DM: *P. gingivalis* and *P. melaninogenica* significantly enriched; *S. mutans* and *S. sobrinus* were not significantly different. No significant saliva or plaque alpha-diversity difference was observed.	The source reports statistically significant enrichment of the two periodontal pathogens but the extracted main text does not provide a single standardized effect size. Salivary cadaverine and L-leucine and plaque N-acetyldopamine and 3,4-dimethylbenzoic acid were significantly higher in T2DM.	Groups did not differ significantly in age or sex, but BMI differed. Strict exclusions removed caries, periodontal and other oral diseases, recent antibiotics/ immunosuppressants and recent periodontal treatment. Analyses used *t*-tests/Wilcoxon tests without multivariable confounder adjustment.
Rahman et al. (53)	Obesity/overweight with periodontal disease Original study	Cross-sectional stratified clinical study Adults aged 18–60 years seeking care at University Dental Hospital Sharjah, UAE Total: 75 participants Groups: Healthy weight *n* = 25; overweight *n* = 25; obesity *n* = 25. Across BMI groups, no-mild periodontitis *n* = 40 and moderate-severe periodontitis *n* = 35.	Pooled subgingival plaque from 8 sites using 16 sterile endodontic paper points after supragingival plaque removal; healthy sulci or deepest periodontal pockets according to status	Full-length (∼1,500 bp) 16S rRNA sequencing with Oxford Nanopore MinION and WIMP/EPI2ME taxonomic workflow	Within moderate-severe periodontitis, *A. actinomycetemcomitans* was enriched in obesity; *T. forsythia* and *T. denticola* in overweight; *P. gingivalis* and *F. nucleatum* in healthy weight. In no-mild periodontitis, *S. enterica* and *K. pneumoniae* were enriched in overweight. These are BMI-by-periodontal-status signatures rather than uniform obesity-related increases.	BMI/body weight correlated positively with probing depth (*P* < 0.01). Alpha and beta diversity differed significantly among stratified groups; LEfSe used LDA-based biomarkers.	Restricted eligibility and excluded recent periodontal/antibiotic treatment, pregnancy, major medical compromise and orthodontic treatment. Clinical and laboratory assessors were blinded. Analyses were stratified by BMI and periodontal severity; no multivariable confounder-adjusted model was reported.
Sun et al. (54)	Ischemic stroke Original study	Observational three-group cross-sectional study with 90-day stroke prognosis follow-up Chinese adults with acute ischemic stroke, individuals with ≥ 3 stroke risk factors, and age/sex-matched healthy controls Total: 146 participants Groups: Ischemic stroke *n* = 52; high-risk ischemic stroke *n* = 48; healthy controls *n* = 46	Morning saliva; participants avoided smoking, gum and mouthwash for 3 h before collection	V3-V4 16S rRNA amplicon sequencing (338F/806R), Illumina MiSeq 2 × 250 bp; QIIME2/DADA2 and LEfSe	*Streptococcus, Prevotella, Veillonella, Fusobacterium* and *Treponema* were more abundant in high-risk and stroke groups than controls. Stroke also showed enrichment of *Bacteroidetes, Fusobacteria* and *Spirochaetes* and progressively higher alpha diversity.	PERMANOVA: HC vs. high-risk *F* = 2.40, HC vs. stroke *F* = 5.07, high-risk vs. stroke *F* = 2.79 (all *P* < 0.001). Microbiome model AUC: 76.3% for high-risk screening; 65.9% for stroke microbiome-only diagnosis; 92.6% for stroke severity; 79.7% (95% CI 64.41–94.97%) for poor 90-day prognosis.	Controls were age- and sex-matched; age, sex and BMI did not differ significantly, but lipid and vascular-risk profiles did. Prediction analyses did not constitute a multivariable causal confounder-adjusted model.
Dai et al. (55)	Metabolic-associated fatty liver disease Original study	Cross-sectional case-control diagnostic modeling study MAFLD patients and healthy volunteers recruited in Shanghai Total: 166 participants Groups: MAFLD *n* = 92; healthy controls *n* = 74; tongue-coating samples *n* = 166 and stool samples *n* = 109	Morning tongue-coating swab scraped from the middle posterior tongue after fasting/rinsing	V3-V4 16S rRNA amplicon sequencing (338F/806R), NovaSeq 6000; QIIME2/DADA2, LEfSe and random forest	Tongue-coating *Streptococcus, Rothia, Neisseria* and *Actinomyces* were identified as MAFLD marker genera; *Streptococcus* and *Rothia* were retained in the combined diagnostic model.	Tongue and gut community beta diversity differed between groups (*P* < 0.001). Combined model using tongue-image features, age/sex/BMI and *Streptococcus*/Rothia achieved 96.39% accuracy.	Groups differed significantly in age, sex, BMI and waist-hip ratio. These variables were incorporated in the prediction model, but differential-abundance results were not reported as confounder-adjusted causal estimates. Major oral and systemic diseases were exclusion criteria.
Chopra et al. (56)	Cardiovascular diseases Secondary metagenomic reanalysis	Systematic dataset identification followed by pooled secondary analysis of six public whole-metagenome studies Public data from adults with atherosclerosis, atrial fibrillation, ischemic attacks and infective endocarditis across five countries Total: 458 metagenomic samples Groups: Gut *n* = 447; cultured blood *n* = 4; arterial plaque *n* = 7	No oral samples; oral origin was assigned by comparison with HOMD/HMP	Reanalysis of paired-end Illumina whole-metagenome FASTQ files using Kaiju v1.9.0; species compared with HOMD and HMP	This analysis cataloged presence rather than case-control increases. Of 17,243 detected microbial species, 410 matched HOMD and occurred in ≥ 1 gut sample; 221 were identified in cultured blood and 169 in plaque. *Streptococcus* and *Rothi*a were among frequently represented oral genera, but a directional CVD association cannot be assigned from this design.	458 samples from six studies; 447 gut, 4 cultured blood and 7 arterial plaque. A subset of 153 species remained oral-associated after HMP filtering.	No participant-level confounder adjustment; heterogeneous diseases and source studies were pooled. Healthy HMP data were used as a filtering comparison.
Lyu et al. (57)	Type 2 diabetes mellitus Mendelian randomization	Bidirectional two-sample Mendelian randomization using summary statistics East Asian oral-microbiome mgGWAS and T2DM GWAS datasets; no newly recruited clinical sample Total: Oral exposure cohort: 2984 healthy Chinese individuals (2017 tongue and 1915 saliva samples). T2DM discovery: 77,418 cases/356,122 controls; replication: 45,383 cases/132,032 controls. Groups: Summary-statistics datasets; no conventional case-control oral sampling groups	Saliva and tongue-dorsum metagenomic traits from the source mgGWAS	mgGWAS-derived species-level genome bins; Wald ratio, IVW, weighted median, MR-Egger, ConMix and MR-RAPS with pleiotropy/heterogeneity and reverse-MR checks	Genetically predicted *Aggregatibacter, Pauljensenia* and *Prevotella* were risk factors for T2DM; *Granulicatella* and *Haemophilus D* were protective. Species within *Catonella, Lachnoanaerobaculum, Streptococcus* and *TM7x* showed mixed directions. These are causal genetic estimates, not observed abundance differences.	Saliva: 23 species were significant in both discovery and replication (12 risk, 11 protective). Tongue analyses also identified replicated associations. Instruments had F-statistics > 10; reverse MR found no T2DM-to-oral-microbiome causal effects (all P > 0.05). Exact species ORs are in supplementary tables rather than the extracted core text.	MR design reduces conventional confounding/reverse causation; analyses included Steiger directionality, Cochran Q, MR-Egger intercept, pleiotropy checks and independent replication. One Haemophilus D analysis showed heterogeneity.
Li et al. (39)	Diabetic coronary heart disease Human clinical cohorts with mouse mechanistic validation	Discovery and independent validation cohorts plus two C57BL/6J mouse experiments testing diabetic myocardial ischemia-reperfusion injury and microbiota manipulation Chinese hospital participants with normal status, diabetes mellitus, coronary heart disease, or diabetic coronary heart disease; 6-week-old male C57BL/6J mice for mechanistic experiments Total: 251 human participants (183 discovery and 68 validation); total number of mice NR because assay-specific N varied Groups: Discovery: NM *n* = 36, DM *n* = 33, CHD *n* = 57, DCHD *n* = 57. Validation: CHD *n* = 33, DCHD *n* = 35. Mouse assay-specific N: generally 4–9 per group as reported in figure legends.	Tongue-coating microbiota	Discovery cohort shotgun metagenomic sequencing on Illumina platforms; Wilcoxon tests with Benjamini-Hochberg FDR correction, Spearman correlations and FEAST source tracking; validation by species/genus-targeted qPCR; mouse antibiotic depletion, Fusobacterium nucleatum gavage or DCHD fecal microbiota transplantation	In DCHD, oral *Fusobacterium nucleatum* increased, whereas *Rothia mucilaginosa, Streptococcus australis* and *Lachnospiraceae bacterium* oral taxon 096 decreased. Gut *Lactobacillus* and *Eubacterium eligens* increased, whereas *Eubacterium* (genus), *E. hallii, Faecalibacterium prausnitzii, E. ramulus, Roseburia faecis* and *E. rectale* decreased. qPCR validated higher oral *F. nucleatum* and higher gut *Lactobacillus* in DCHD versus CHD.	Discovery oral/gut community states differed across groups (Bray-Curtis *P* < 0.001). Oral F. nucleatum correlated positively with gut Lactobacillus (*P* < 0.05). The best single DCHD discriminator was *F. nucleatum* (AUC 0.756); oral *F. nucleatum* plus gut *Eubacterium* intestinalis reached AUC 0.838. Validation showed higher oral *F. nucleatum* and gut *Lactobacillus* in DCHD versus CHD (both *P* < 0.05). In mice, *F. nucleatum* gavage increased infarct area versus antibiotic-pretreated controls (*P* < 0.05), supporting experimental validation beyond the clinical associations.	Clinical comparisons used FDR-corrected differential-abundance tests and summarized cardiovascular risk factors and medications; the validation CHD and DCHD groups had no significant differences in most demographic, medication and laboratory variables, although metformin and glycemic indices differed by disease definition. No multivariable covariate-adjusted microbiome model was reported. Mouse experiments used controlled housing and intervention groups.
Leonov et al. (58)	Obesity and cardiovascular risk Cross-sectional human study	Cross-sectional age- and BMI-stratified study conducted from 2020 to 2023 189 Caucasian adults examined at a Russian nutrition clinic, divided into young and older obese and non-obese groups Total: 189 Groups: Young obese *n* = 57; young control *n* = 48; older obese n = 57; older control *n* = 27	Pooled supragingival/tooth-surface biofilm swabs and unstimulated saliva	Species-specific PCR/qPCR targeting Porphyromonas gingivalis, Prevotella intermedia, Aggregatibacter actinomycetemcomitans, Tannerella forsythia, Treponema denticola and Fusobacterium nucleatum	Periodontal-pathogen detection varied by age and obesity rather than representing global microbiome abundance. *P. intermedia* was more prevalent in young obese than young control participants; *A. actinomycetemcomitans* was more prevalent in older obese than older controls; *T. denticola* was more prevalent in obese groups at both ages and was strongly age-associated. *F. nucleatum* was detected in nearly all samples and was independent of age or weight. *P. gingivalis* showed an age-related increase and a nonsignificant obesity trend in older adults.	Overall prevalence: *P. gingivalis* 33.0%, P. intermedia 47.8%, A. actinomycetemcomitans 63.4%, T. forsythia 46.6%, *T. denticola* 46.6% and *F. nucleatum* 89.2%. P. intermedia prevalence was 49.1% versus 26.1% in young obese versus controls (*P* = 0.019); A. actinomycetemcomitans was 78.6% versus 46.2% in older obese versus controls (*P* = 0.004); *T. denticola* was 50.0% versus 22.7% in young groups (*P* = 0.007) and 67.9% versus 34.6% in older groups (*P* = 0.005). *P. intermedia* was associated with lower HDL and higher LDL/HDL and atherogenic index; *T. forsythia* with higher triglycerides and atherogenic index; *T. denticola* with higher relative CVD risk.	Participants were stratified by age and BMI. Analyses used Mann-Whitney, chi-square and Tau-b Kendall correlations, without a reported multivariable covariate-adjusted model. Cardiovascular and diabetes risks were assessed using SCORE and FINDRISC.
Mohammed et al. (59)	Smoking exposure and lipid biomarkers Cross-sectional human study	Cross-sectional comparison of self-reported smokers and non-smokers selected from Qatar Biobank 300 Qatari biobank participants selected irrespective of age and health status Total: 300 enrolled; 244 retained for microbiome analyses after read-quality filtering Groups: Initial: smokers *n* = 200 and non-smokers *n* = 100. After filtering for relative-abundance analyses: smokers *n* = 144 and non-smokers *n* = 100.	Whole saliva collected by direct spitting	16S rRNA V3-V4 amplicon sequencing; QIIME2/DADA2 and SILVA 138 taxonomy; Wilcoxon tests, PERMANOVA, Benjamini-Hochberg correction and Spearman correlations	Smokers had increased *Streptococcus*, including *Streptococcus salivarius*, and decreased *Porphyromonas, Neisseria, Veillonella* and *Fusobacterium* relative to non-smokers. *Streptococcus* abundance was positively correlated with LDL and negatively correlated with HDL; these are exposure-associated correlations, not evidence that smoking-induced taxa caused lipid changes.	*Streptococcus* mean relative abundance was 39.20% in smokers versus 12.40% in non-smokers (3.08-fold, BH-adjusted *P* = 5.53 × 10^–24^). *S. salivarius* averaged 14% in smokers and was not detected in non-smokers. *Porphyromonas, Neisseria* and *Veillonella* were reduced 6. 32-, 9. 57-, and 1.56-fold, respectively; *Fusobacterium* was 0% versus 0.65%. Smokers also had higher triglycerides (1.37 versus 1.17 mmol/L, *P* = 0.033), higher LDL (3.23 versus 2.85 mmol/L, *P* = 0.0045) and lower HDL (1.28 versus 1.45 mmol/L, *P* = 0.000639).	Microbial comparisons used BH-adjusted P values and Spearman correlations. No multivariable adjustment was reported. Smokers were older and differed in triglycerides, LDL and HDL, so lipid-microbiome findings should be interpreted as correlations rather than causal effects.
Bai et al. (60)	High-fat-diet-induced obesity Animal experiment	Randomized controlled high-fat-diet versus normal-chow mouse experiment over 20 weeks Five-week-old specific-pathogen-free male C57BL/6 mice Total: 55 mice Groups: Normal-chow diet *n* = 25; high-fat diet *n* = 30	Salivary/oral-area swab samples	16S rRNA V3-V4 amplicon sequencing on Illumina NovaSeq 6000; QIIME2/Greengenes2, diversity analyses, LEfSe and PICRUSt2; fecal SCFA/MCFA measurement by GC-MS/MS	High-fat diet increased salivary *Streptococcus* and *Escherichia*. Across both salivary and gut microbiota, *Lactobacillus* and *Romboutsia_B* increased, whereas seven genera including *Akkermansia, Muribaculum* and *Intestinimonas* decreased. Gut-specific HFD enrichment included *Kineothrix* and *Cryptobacteroides*, with reduced CAG-485.	Salivary beta diversity differed between HFD and NCD groups (*P* = 0.011), and gut beta diversity showed stronger separation (*P* = 0.001). LEfSe identified 10 salivary taxa enriched under HFD and 46 under NCD; 38 gut taxa were enriched under HFD and 62 under NCD. Lactobacillus correlated positively with TC, LDL and glucose in gut samples and with HDL, LDL and TC in salivary samples. HFD mice also had significantly higher serum TC, TG, HDL-C and LDL-C than NCD mice.	Mice were randomly allocated; groups were housed separately under controlled conditions, samples were collected at the same time of day and sequenced in the same run. Analyses used Bonferroni or Dunn multiple-comparison procedures as appropriate; no observational covariate adjustment was applicable.
Takagi et al. (61)	Dyslipidemia Community-based longitudinal cohort	Community health-check cohort with a 2017 cross-sectional analysis and 2017–2019 longitudinal dyslipidemia-onset analysis 763 adult residents participating in the Iwaki Health Promotion Project in Japan in both 2017 and 2019 Total: 763 Groups: 2017: normal lipids *n* = 541, dyslipidemia *n* = 222. 2019: normal *n* = 524, dyslipidemia *n* = 239. Longitudinal onset analysis among participants normal in 2017: remained normal *n* = 464, developed dyslipidemia *n* = 77.	Self-collected dorsal tongue-coating swab immediately after waking	16S rDNA V3-V4 amplicon sequencing on MiSeq; DB-BA taxonomy; Bray-Curtis PERMANOVA, LEfSe and multiple logistic regression	Cross-sectionally, dyslipidemia was associated with higher *Tannerella, Treponema, Veillonella, Atopobium, Megasphaera* and *Stomatobaculum*, whereas *Haemophilu*s and *Neisseria* predominated in controls. Longitudinally, only higher *Megasphaera* abundance remained significantly associated with new-onset dyslipidemia in the adjusted model.	Cross-sectional oral-community composition differed between normal and dyslipidemia groups in 2017 (*P* = 0.042, *R*^2^ = 0.0024). Among 541 participants normal in 2017, 77 developed dyslipidemia and 464 remained normal; longitudinal beta diversity differed (*P* = 0.037, *R*^2^ = 0.0047). Adjusted Megasphaera association with dyslipidemia onset: OR 1.005 per myriad relative-abundance unit, 95% CI 1.000-1.009, *P* = 0.038.	Multiple logistic regression adjusted for age, sex, BMI and alcohol intake. Participants with missing data, antibiotic use or cancer history were excluded.

Study designs, populations, oral sampling sites, analytical methods, principal taxonomic findings, quantitative results, and confounder handling are summarized from the verified structured dataset. Taxonomic ranks and unresolved limitations are retained as reported; NR indicates not reported.

When interpreting oral microbiome findings across GLMD-related conditions, several methodological factors should be considered. Current studies differ substantially in oral sampling sites, including saliva, tongue coating, supragingival plaque, subgingival plaque, and oral swabs. These niches harbor distinct microbial communities and therefore cannot be interpreted interchangeably. In addition, study populations vary in age, ethnicity, dietary habits, smoking status, oral hygiene, periodontal status, medication exposure, and metabolic disease severity. Sequencing platforms, taxonomic resolution, reference databases, and bioinformatic pipelines also differ across studies. Therefore, recurrent taxa across diseases should be interpreted as potentially informative signals, whereas inconsistent or contradictory findings may reflect methodological heterogeneity, population differences, or disease-specific biology rather than true biological conflict.

Taxa were included if they met at least one of the following criteria: they were reported in at least two disease categories; they represented discordant association directions within the same disease; or they were cross-disease taxa highlighted in this Review. Arrows indicate enrichment or positive association (↑), depletion or inverse association (↓), and discordant directions (↕); question marks indicate insufficient evidence to assign a consistent direction. Blank cells indicate that no association was identified in the included literature. Genus- and species-level findings are presented separately. Bars indicate the number of disease categories in which each taxon was reported and do not represent evidence strength. These associations are descriptive and should not be interpreted as evidence of causality.

### 4.1 T2DM

In T2DM, shifts in the oral microbiota have been observed. Earlier work often attributed microbiota changes in metabolic disease to underlying oral pathology. However, Li et al. showed that, even in T2DM patients without evident oral disease, both oral microbial composition and metabolite differed from those of healthy controls (52). In these patients, *P. gingivalis* and *Prevotella melaninogenica* were significantly enriched, while levels of caries-associated species such as *Streptococcus mutans* and *Streptococcus sobrinus* were similar to those in controls (52). This work provided early evidence that oral microbial changes can occur in T2DM even in the absence of local oral pathology.

Genetic analyses suggest an association between certain oral microbes and T2DM. A bidirectional two-sample Mendelian randomization (MR) study identified several oral genera that may increase the risk of T2DM. *Aggregatibacter, Pauljensenia*, and *Prevotella* were genetically associated with higher T2DM risk, whereas *Granulicatella* and *Haemophilus D* were genetically associated with lower T2DM risk (57). These MR findings provide genetic evidence suggesting potential causal associations between selected oral genera and T2DM, but they should be interpreted cautiously because MR results depend on instrument validity, population specificity, and taxonomic resolution. A separate study by Chen and colleagues profiled the oral microbiota of Chinese adults with T2DM using 16S rRNA sequencing (49). They reported a higher *Firmicutes/Bacteroidetes* ratio. Some genera were more abundant, including *Neisseria, Streptococcus*, and *Haemophilus*, while *Acinetobacter* trended lower. The discrepancies among T2DM studies may partly arise from differences in sampling and analytical strategy. Li et al. analyzed both saliva and supragingival plaque by metagenomics and untargeted metabolomics in T2DM patients without oral diseases (52), whereas Chen et al. collected oral swabs from Chinese T2DM patients and controls and analyzed the V1–V2 region of 16S rRNA using QIIME (49). Therefore, differences in oral niche, ethnic background, sequencing depth, taxonomic resolution, and bioinformatic pipelines may explain why some studies emphasize *P. gingivalis* and *Prevotella* melaninogenica, while others identify *Neisseria, Streptococcus, Haemophilus*, or *Acinetobacter*.

Alterations in the oral microbiome are detectable during early metabolic dysregulation. Studies have reported lower salivary microbial richness in people with prediabetes, accompanied by a tendency toward decreased *Streptococcus* abundance (62). Analyses of tongue-coating samples from people with PreDM have also noted altered levels of *Corynebacterium* and *Johnsonella* (63). Examining these early shifts may provide clues to the progression from prediabetes to diabetes.

Glycemic control and diabetes duration are associated with changes in the oral microbiota. In the elderly with T2DM, poorer fasting glucose control often coincides with shifts in the oral microbiota. Taxa such as *Leptotrichia*, *Staphylococcus*, *Catonella*, and *Bulleidia* tend to appear in higher abundance under these conditions (47). Longer disease courses are often associated with higher *Streptococcus* levels, whereas *Cardiobacterium* level tends to decrease (64). Most studies report links between glycemic control, disease duration, and shifts in the oral microbiota. A few smaller studies, however, did not find clear associations between HbA1c and oral microbial profiles (64). Taken together, these observations indicate that the oral microbiota may reflect poor glucose regulation and may have potential as a candidate biomarker, although its relationship with disease progression remains to be validated longitudinally. Current evidence remains mainly associative and cannot determine whether these microbial shifts precede or result from impaired glucose metabolism.

Antidiabetic therapies appear to change the oral microbiome. Comparisons among non-diabetic individuals, newly diagnosed patients, and those treated with metformin or insulin-based regimens revealed changes in multiple taxa. Among the genera that differed were *Blautia wexlerae*, *Lactobacillus fermentum*, *Nocardia coeliaca*, and *Selenomonas artemidis* (65). Dietary intervention produced a different pattern. In one study of individuals with T2DM, the gut microbiota responded more visibly to dietary modification than the oral microbiota. The oral microbiome was more dynamic at the genetic-strain level. Strain-level variation may be involved in diet-associated changes in glycemic, metabolic, and immune measures (65, 66).

### 4.2 Fatty liver

NAFLD is regarded as the most common chronic liver disease worldwide (67). As the disease advances, some individuals develop nonalcoholic steatohepatitis (NASH), a stage marked by inflammation and fibrosis that raises the likelihood of cirrhosis and hepatocellular carcinoma (HCC). The terminology surrounding this condition has shifted over the past few decades. Since Schaffner and Thaler first introduced the term “NAFLD” in 1986 (68), it has been widely used in clinical practice and research. However, in 2020, an international expert consensus published in *Gastroenterology* proposed the name metabolic dysfunction-associated fatty liver disease (MAFLD) to reflect better the central role of metabolic dysfunction (69). More recently, at the 2023 European Association for the Study of the Liver Annual Meeting, experts further recommended adopting the term metabolic dysfunction–associated steatotic liver disease (MASLD) (70). According to the working groups, the MAFLD definition may better identify individuals at elevated risk of cardiovascular disease (CVD) events (71), even after adjustment for conventional risk factors (72).

Growing evidence links microbial communities with NAFLD and NASH-related pathological features. Although much microbiome research in fatty liver disease has focused on the gut, oral microbial alterations have attracted increasing attention. Researchers have proposed the concept of the “oral–liver axis,” emphasizing the biological connection between the oral microbiome and liver diseases (73). A systematic review reported associations between oral microbiota profiles and NAFLD, with specific taxa indicating higher disease risk (74). At the higher taxonomic level, studies reported differences in the abundance of *Firmicutes*, *Proteobacteria*, and *Clostridia* compared with healthy controls. At the genus and species levels, *Porphyromonas*, particularly *P. gingivalis*, has been repeatedly associated with NAFLD-related pathological features. Some studies have proposed that the *P. gingivalis/Porphyromonas* ratio may serve as a potential marker of NAFLD progression. A 2022 study detected *Porphyromonas gingivalis* in liver tissue and showed in experimental models that its LPS can promote features consistent with NAFLD progression in experimental settings (37). The organism also influences gut microbial structure, and these disturbances may aggravate liver injury under experimental or dysbiotic conditions by altering metabolic and immune processes. Endotoxemia related to *P. gingivalis* has therefore been considered a plausible contributor to NAFLD-related inflammatory and metabolic disturbance.

Tongue coating microbiota are associated with coating phenotype in MAFLD patients. Lu et al. collected tongue coating and stool samples from 38 MAFLD patients and used full-length 16S rDNA assembly sequencing to compare white and yellow tongue-coating clusters. They reported that *Flavobacteriia* exhibited significantly higher abundance in the yellow coating group than the white coating group. Additionally, *Mollicutes*, *Deltaproteobacteria, Corynebacterium, Moraxella, Ottowia, Lactobacillus, Johnsonella, Tissierella*, and *Enterobacter* were unique to the white coating group, whereas *Shuttleworthia, Simonsiella, Desulfobulbus*, and *Mycoplasma* were specific to the yellow coating group. In terms of relative abundance, *Neisseria* predominated in the white coating, while *Prevotella* was dominant in the yellow coating (30). These findings suggest that tongue appearance often reflects underlying microbial differences. Furthermore, Dai et al. explored the application of tongue coating microbiota and tongue appearance in diagnosing MAFLD (55), identifying *Streptococcus, Rothia, Neisseria*, and *Actinomyces* as potential biomarkers in 92 MAFLD patients. They developed a diagnostic model integrating tongue appearance, basic patient information (sex, age, BMI), and marker bacteria (*Streptococcus, Rothia*) that achieved an accuracy of 96.39%, higher than models that excluded microbial data. However, these tongue-coating findings may not be directly comparable with studies focusing on periodontal taxa or whole-oral microbial profiles, because tongue coating represents a specific dorsal-tongue biofilm niche. Differences in NAFLD, MAFLD, and MASLD definitions, obesity status, diet, and periodontal inflammation may also contribute to inconsistent microbial signatures.

On the other hand, the oral microbiome is influenced by multiple factors, including diet, stress, tobacco use, and systemic health. Lifestyle factors are associated with differences in oral microbial composition and may partly explain inter-individual variation in MAFLD-related oral microbial profiles (74). For example, structured exercise can enhance oral microbial diversity and reduce both the abundance and pathogenic potential of lipopolysaccharide (LPS)-producing periodontal bacteria, thereby improving the oral environment of NAFLD patients (75). Moreover, *Nisin lantibiotic* has been reported to prevent hepatic steatosis and mitochondrial oxidative stress in NAFLD following periodontal disease by mitigating dysbiosis in the oral cavity, gut, and liver (76). These findings suggest that treatment-related microbial changes may accompany metabolic improvement, although mediation has not been established.

### 4.3 Obesity

Growing evidence indicates that obesity is associated with oral microbial dysbiosis. In adults, children, and animal models, obesity is accompanied by marked remodeling of microbial community structure, dysfunction, and an increased risk of inflammation.

In adults, the oral microbiota of obese individuals shows significant differences in diversity, composition, and function. Studies have found that the salivary microbiota of obese individuals exhibits lower diversity and richness, with specific genera such as *Prevotella, Granulicatella*, and *Peptostreptococcus* significantly increased, whereas *Haemophilus* and *Corynebacterium* decreased (45). This salivary pattern was derived from periodontally healthy adults with obesity and normal-weight controls, using V3–V4 16S rRNA sequencing on saliva samples. It may therefore represent obesity-associated salivary dysbiosis in the absence of overt periodontal disease. By contrast, Rahman et al. analyzed subgingival plaque from healthy-weight, overweight, and obese participants further stratified by no-mild or moderate-severe periodontitis, using nanopore 16S rRNA sequencing (53). Obese subjects showed enrichment of highly pathogenic bacteria in the subgingival microbiota, including *Aggregatibacter actinomycetemcomitans*, *Tannerella forsythia*, and *P. gingivalis*. The enrichment of *A. actinomycetemcomitans, T. forsythia*, and *P. gingivalis* in this study may therefore reflect the combined influence of body weight and periodontal inflammation rather than adiposity alone. These findings suggest that obesity is associated with altered microbial community structure and may interact with periodontal inflammatory status.

Functional analyses identified predicted metabolic features associated with obesity. In a cohort of diabetic patients from Qatar, the *Firmicutes/Bacteroidetes* ratio was significantly elevated in obese individuals (77). Liang et al. reported that obesity alters the structure of the salivary microbiota and that pathways related to biofilm formation are markedly upregulated, suggesting that these bacterial communities may possess stronger adhesion and immune evasion capabilities. In addition, microbial network analysis identified *Bergeyella* as a potential bridging genus, suggesting a possible role in community organization (78). Similarly, J. M. Goodson et al. found that the salivary microbial composition differed significantly in overweight women (42). Classification tree analysis of salivary microbiological composition revealed that 98.4% of the overweight women could be identified by the presence of a single bacterial species (*Selenomonas noxia*) at levels greater than 1.05% of the total salivary bacteria. This species appears to be a potential biomarker of overweight status.

Childhood obesity is likewise accompanied by marked alterations in the oral microbiome, characterized by early dysbiosis in microbial diversity, composition, and metabolic function. In obese children, salivary microbial α-diversity is increased, with significantly higher abundances of *Haemophilus*, *Aggregatibacter*, and *Actinobacillus* (78). A study of children aged 3–5 years further revealed that the *Firmicutes/Bacteroidetes* (F/B) ratio was significantly elevated in both the oral and gut microbiota of obese children (79). At the genus level, *Filifactor* and *Butyrivibrio* were markedly enriched, reflecting a microbial profile associated with obesity. Evidence from a systematic review supports this trend. A meta-analysis of 11 studies demonstrated consistent increases in dominant phyla such as *Firmicutes*, *Proteobacteria*, and *Actinobacteria* among overweight and obese children (80).

The oral environment of obese children shows characteristics of inflammation and metabolic dysregulation. Multi-omics analyses have shown that the levels of inflammatory cytokines (e.g., TNF-α, IL-1β) and metabolic metabolites (e.g., glutamate, cholesterol) in the saliva of children with obesity or metabolic syndrome were significantly elevated, and the health-associated genera such as *Streptococcus* and *Actinomyces* were reduced. These findings are consistent with an oral inflammatory and metabolic-risk phenotype in obese children, rather than proving that oral dysbiosis initiates obesity-related metabolic abnormalities (81). Notably, such microbial alterations may originate as early as the maternal stage. Seifert et al. reported that maternal obesity significantly alters the initial oral microbiota of neonates, with an increased proportion of *Peptoniphilus* species, and is associated with an increased risk of subsequent infections, allergies, and immune-related disorders (82). This observation suggests that obesity-associated microbial differences may emerge as early as the maternal-infant period, although longitudinal studies are needed to determine persistence and clinical consequences.

Animal studies have also provided evidence for the relationship between obesity and the oral microbiome. In a mouse model fed a high-fat diet (HFD), salivary abundances of *Streptococcus* and *Escherichia* were significantly increased, whereas *Rodentibacter* predominated in the control group (60). In addition, nine bacterial genera changed simultaneously in the oral and gut microbiota, supporting coordinated oral-gut microbial responses to high-fat diet in this animal model. Among these, *Akkermansia*, *Lactobacillus*, and *Intestinimonas* were closely related to physiological indicators of obesity.

Clinical interventions that improve obesity also modulate the oral microbiota. Bariatric surgery has been shown to markedly reshape the oral microbial ecosystem, with a gradual shift toward a healthier composition. *Streptococcus salivarius* and several *Veillonella* species significantly increased, and periodontal pathogenic clusters containing *Porphyromonas* spp. were substantially reduced (83).

Studies have shown that obesity is closely associated with oral microbial diversity, pathogenic enrichment, and functional imbalance. Meanwhile, dietary patterns, ethnic background, and maternal conditions may directly or indirectly influence obesity risk and metabolic health by modulating microbial composition (82, 84). These observations highlight the oral microbiome as a potential mediator or modifiable microbial feature associated with obesity.

### 4.4 Dyslipidemia

There is a potential association between dyslipidemia and the oral microbiota, as oral microbial communities may reflect lipid metabolic status and may be linked to lipid-related inflammatory or metabolic pathways.

In subgingival plaque samples of people with metabolic disorders, a significant positive correlation was observed between elevated triglyceride levels (>1.7 mmol/L) and the relative abundance of *Actinomyces naeslundii*, *Actinomyces odontolyticus*, *Aggregatibacter segnis*, *Corynebacterium matruchotii*, *Fusobacterium canifelinum*, *Fusobacterium nucleatum*, *Fusobacterium periodonticum*, and *Streptococcus sanguinis*. Positive associations between low high density lipoprotein (HDL) levels (≤1.0 mmol/L in men and ≤1.3 mmol/L in women) and *Actinomyces naeslundii*, *Actinomyces odontolyticus*, *Aggregatibacter segnis*, *Corynebacterium matruchotii*, *Fusobacterium periodonticum*, and *Haemophilus parainfluenzae* were also reported (51). Because these associations were identified from subgingival plaque in a South African cohort with and without metabolic syndrome, they may be strongly influenced by periodontal ecology and low-grade inflammatory status. Another study demonstrated that serum concentrations of total cholesterol (TC), low density lipoprotein (LDL), and triglycerides (TG) were positively correlated with *P. gingivalis*, while *Tannerella forsythia* was associated with LDL, and *Treponema denticola* with TG levels (58).

This differs from the large Japanese community-based study, which used tongue-coating samples from 763 health-checkup participants and 16S rDNA amplicon sequencing. They identified specific genera strongly associated with dyslipidemia, including *Veillonella*, *Atopobium*, *Stomatobaculum*, *Tannerella*, and *Megasphaera* (61). The identification of *Megasphaera* and other tongue-coating genera may therefore reflect a dorsal-tongue microbial niche rather than the periodontal plaque-associated taxa observed in subgingival studies.

Smoking is another major source of heterogeneity. Mohammed et al. profiled salivary microbiota from 200 smokers and 100 non-smokers in a Qatari population using V3–V4 16S rRNA sequencing and QIIME-based OTU analysis. In particular, *Streptococcus* showed a significant positive correlation with LDL and a negative correlation with HDL (59). Therefore, lipid-associated *Streptococcus* signals in this setting should be interpreted together with smoking-induced salivary dysbiosis.

Lipid-lowering interventions and medication exposure may also influence oral microbial profiles and should be considered when interpreting dyslipidemia-associated microbial signatures. Statins, in addition to reducing serum cholesterol levels, inhibit *P. gingivalis* growth and markedly decrease bacterial accumulation in both newly formed and mature biofilms, may act as a confounder or modifier in oral microbiome studies of dyslipidemia (48). So drug exposure further complicates interpretation, because statins may directly inhibit *P. gingivalis* biofilm formation, making it difficult to distinguish lipid-status associations from medication-induced microbial changes.

### 4.5 Hypertension

There is an association between hypertension and the oral microbiota. Increasing evidence indicates that individuals with hypertension exhibit distinct differences in oral microbial diversity, composition, and ecological stability compared with normotensive individuals. In a prospective cohort of 1,215 postmenopausal women followed for an average of 10.4 years, fifteen specific oral bacterial taxa were found to be significantly associated with the incidence of hypertension. Ten taxa (*Streptococcus anginosus*, *Streptococcus salivarius*, *Fretibacterium* sp. oral taxon 362, *Selenomonas infelix*, *Prevotella* sp. oral taxa 526, 292, and 376, *Megasphaera* sp. oral taxon 123, *Capnocytophaga* sp. oral taxon 903, and *Streptococcus lactarius*) showed positive associations, and five (*Neisseria subflava*, *Bergeyella* sp. oral taxon 907, *Gemella morbillorum*, *Leptotrichia* sp. oral taxon 212, and *Aggregatibacter segnis*) showed negative associations. These correlations remained robust after adjustment for demographic, clinical, and lifestyle confounders, providing prospective observational evidence at the population level, although they do not by themselves establish causality (50).

Further clinical and experimental studies demonstrated that hypertensive individuals exhibit significantly reduced α-diversity and altered β-diversity in their oral microbiota. Salivary and subgingival *Porphyromonas*, *Fusobacterium*, and *Treponema* species were positively correlated with serum inflammatory factors, including IL-6 and CRP (40). Animal studies further showed that the blood pressure of mice transplanted with hypertensive saliva was significantly increased, and oral-derived *Veillonella* may ectopically colonize the gut and aggravate hypertension. The oral-gut transmission study provides mechanistic support but also highlights the role of periodontal status: it collected saliva, subgingival plaque, and fecal samples, included cross-sectional and 6-month follow-up cohorts, and found higher blood pressure in hypertensive participants with periodontitis.

Oral nitrate-reducing bacteria may contribute to blood pressure regulation through the nitrate-nitrite-NO pathway. Nitric oxide (NO) exerts potent vasodilatory and antihypertensive effects (85). Certain bacteria, such as *Prevotella melaninogenica* and *Veillonella dispar*, can reduce dietary nitrate to nitrite and further generate NO, thereby maintaining host NO homeostasis (46). Conversely, a lower abundance or activity of nitrate-reducing bacteria may reduce NO bioavailability and is associated with higher blood pressure. Clinical evidence shows that patients with hypertension had significantly lower plasma NO levels and decreased abundances of nitrate reducing bacteria such as *Neisseria subflava* and *Veillonella* (86, 87). If antibacterial mouthwash is used to inhibit such bacteria, NO production is reduced, and blood pressure increases (86, 87), supporting the physiological relevance of oral nitrate-reducing bacteria in blood pressure regulation.

For hypertension, the most persuasive evidence is functional rather than purely taxonomic. Studies measuring tongue scraping, saliva, plasma nitrate/nitrite, oral nitrate-reducing capacity, and blood pressure response after nitrate supplementation show that diet-derived nitrate, mouthwash use, oral hygiene, sex, pregnancy status, and antihypertensive treatment may all modify oral microbial effects. Recent clinical intervention studies have revealed that supplementation with nitrate-rich leafy vegetables or potassium nitrate improved oral microbial structure, enhanced NO bioavailability, and reduced blood pressure (88, 89). The magnitude of the blood pressure lowering effect was strongly associated with the abundance and activity of oral nitrate-reducing bacteria, with higher levels observed in better responders (88). Similarly, Chen et al. found that antihypertensive efficacy was associated with the tongue-coating microbiota and could even predict the direction of individual responses (90). The effects of mouthwash intervention on blood pressure in healthy subjects with different tongue coating phenotypes were completely different. Participants with a “yellow greasy coating” showed a significant reduction in blood pressure (approximately -9 mmHg systolic and -3 mmHg diastolic). In comparison, those with a “white coating” exhibited the opposite trend (approximately + 8 mmHg systolic). These findings raise the possibility that oral microbial features may help stratify individual responses to dietary or oral ecological interventions, although standardized response definitions and prospective validation are still needed.

### 4.6 Cardiovascular disease

CVD is the leading cause of mortality and disease burden worldwide, and disturbances in the oral microbiota have been associated with cardiovascular events and may participate in related inflammatory and metabolic pathways. These mechanisms include oral and systemic inflammation, immune dysregulation, cytokine release, bacteremia, microbially derived metabolic and signaling pathways involving SCFAs, TMAO, hydrogen sulfide (H_2_S), and NO, as well as specific microbial toxins such as LPS and leukotoxin A (LtxA) (91). These pathways provide plausible biological links between oral microbial imbalance and atherosclerosis, coronary artery disease, and heart failure.

Oral microbes and their components may reach the bloodstream through barrier disruption or transient bacteremia, and their detection in vascular plaques supports a plausible link between oral microbial dissemination and cardiovascular pathology. As early as 2005, scholars found that live oral pathogens can be detected in the vascular plaques of patients with atherosclerosis, including *P. gingivalis* and *Aggregatibacter actinomycetemcomitans* (41). Subsequent research has identified a variety of oral microbes in atherosclerotic lesions, including *Fusobacterium nucleatum* (Fn), *Prevotella intermedia* (Pi), *Tannerella forsythia* (Tf), *Treponema denticola* (Td), and *Campylobacter rectus* (Cr) (92, 93). These studies differ from saliva-only biomarker studies because they directly examined potential microbial translocation routes. Koren et al. used 454 pyrosequencing to compare atherosclerotic plaque, oral, and gut samples from patients with atherosclerosis and found that *Veillonella* and *Streptococcus* in plaques correlated with their oral abundance (43). Chopra et al. further used whole-metagenomic data to search for oral bacteria in gut, arterial plaque, and cultured blood samples according to the Human Oral Microbiome Database (56), such as *Escherichia*, *Prevotella*, *Bacteroidetes*, *Lactobacillus*, *Ruminococcus*, *Eubacterium*, and *Streptococcus*. Such multi-site evidence provides stronger biological plausibility for oral-derived microbial dissemination, although it still cannot prove causality.

Elevated levels of *Aggregatibacter actinomycetemcomitans* in the oral cavity are significantly associated with the risk of coronary artery disease (CAD) and acute coronary syndrome (ACS), and the risk increased with every 10-fold increase in saliva concentration (44). Another study reported that high salivary abundance of *Streptococcus* and *Rothia* is associated with myocardial infarction risk, and molecular traces of these taxa were detected in the bloodstream, indicating that the oral microbiota may spread via the bloodstream and affect myocardial function. Compared with the gut microbiota, the oral microbiome has higher activity and greater migratory potential, which may disrupt immune homeostasis and trigger inflammatory responses that are implicated in atherosclerosis (56). Therefore, alterations in oral microbial composition may serve as candidate biomarkers for CVD risk assessment, but their predictive value requires validation in independent prospective cohorts.

These associations have prompted investigation of clinical utility. Researchers have further investigated the diagnostic and predictive value of oral microbiota across different cardiovascular disease subtypes. In patients with diabetes and coronary heart disease (DCHD), oral *Fusobacterium nucleatum* levels are significantly elevated and show co-enrichment with gut taxa such as *Lactobacillus*. This pattern may serve as a target for predicting and intervening in DCHD (39). In another study, oral *F. nucleatum* was again identified as a potential microbial biomarker for DCHD, and animal experiments suggested that *F. nucleatum* expansion may aggravate myocardial injury under hyperglycaemic conditions (39). In contrast, salivary case-control studies and machine-learning models may be useful for biomarker discovery but are more sensitive to cohort composition and analytical pipelines. For example, Kato-Kogoe et al. analyzed saliva from 43 ACVD patients and 86 age- and sex-matched controls using 16S rRNA sequencing and random forest modeling with 10-fold cross-validation. The overall β-diversity of the oral microbiota is markedly altered, characterized by increased abundance of *Actinobacteria* and decreased *Bacteroidetes*. A machine learning model achieved an area under the curve (AUC) of 0.933 in the validation set, showing promising discrimination in an internally validated dataset (94). These results support diagnostic feasibility, but they should be interpreted separately from plaque- or blood-based evidence of microbial translocation. Besides, in ischemic stroke (IS) patients, the relative abundances of *Streptococcus*, *Prevotella*, *Veillonella*, *Fusobacterium*, and *Treponema* are increased. Models constructed from these microbial differences effectively predict 90-day patient prognosis (54), which further suggests the feasibility of the oral microbiota as a biomarker.

Overall, CVD studies also face strong confounding from age, smoking, diabetes, dyslipidemia, hypertension, periodontal disease, diet, and cardiovascular medications. Therefore, the strongest current evidence supports an association among oral dysbiosis, systemic inflammation, microbial translocation, and vascular risk, whereas the causal role of specific oral taxa in distinct cardiovascular events remains uncertain.

### 4.7 Interpreting oral microbiome evidence in GLMD

Taken together, studies across T2DM, fatty liver disease, obesity, dyslipidemia, hypertension, and cardiovascular disease suggest that oral microbial alterations are associated with multiple GLMD-related conditions. However, these associations require critical interpretation because oral microbiome profiles are strongly shaped by oral health status, host exposures, sampling strategies, analytical pipelines, and study design. Therefore, before moving from disease-associated taxa to mechanistic or translational conclusions, it is necessary to distinguish confounding and heterogeneity from causal relevance.

#### 4.7.1 Confounding and heterogeneity in reported microbial associations

A major challenge in interpreting oral microbiome signatures in GLMD is that many reported taxa are not specific to metabolic pathology. The oral microbiome is highly sensitive to local ecological conditions, including periodontal inflammation, plaque accumulation, caries status, tongue coating, salivary flow, oral hygiene behavior, mouthwash use, smoking, diet, age, and medication exposure. These factors may independently alter oral microbial communities. Therefore, a microbial pattern observed in GLMD patients may reflect metabolic dysfunction, oral-health-related dysbiosis, host exposure, or a combination of these processes.

Periodontal status is the most important confounder because many recurrent GLMD-associated taxa are also classical periodontal or oral inflammatory taxa. *Porphyromonas, Fusobacterium, Prevotella, Tannerella, Treponema*, and *Aggregatibacter* frequently appear across studies of diabetes, obesity, dyslipidemia, hypertension, and cardiovascular disease, but these taxa are also strongly linked to periodontal biofilms and gingival inflammation. The original studies illustrate how strongly study design affects interpretation. Some studies attempted to reduce oral-health-related bias by excluding participants with overt oral or periodontal diseases. For example, Li et al. analyzed saliva and supragingival plaque from T2DM patients without overt oral diseases (52), Wu et al. excluded participants with systemic, oral mucosal, or periodontal diseases when studying salivary microbiota in adults with obesity (45), and Salman et al. studied periodontally healthy, caries-free children with obesity/metabolic syndrome using unstimulated saliva and multi-omics analysis (81). These designs make it more plausible that observed microbial differences are related to metabolic status, although they do not fully eliminate residual effects of plaque accumulation, oral hygiene, or niche-specific sampling. In contrast, studies that directly included periodontal assessment show how easily metabolic and periodontal signals can overlap. Rahman et al. analyzed subgingival plaque in overweight and obese individuals stratified by periodontal status and found that periodontal pathogens such as *Aggregatibacter actinomycetemcomitans, Tannerella forsythia*, and *Porphyromonas gingivalis* were associated with both body-weight category and periodontitis (53). Similarly, Chen et al. investigated oral-gut microbial transmission in hypertension using saliva, subgingival plaque, and fecal samples and found that hypertensive participants with periodontitis had higher blood pressure (40). Therefore, when certain oral microbial taxa are reported to be associated with GLMD, it should first be considered whether they are oral-health-related microbial groups, unless periodontal status and oral hygiene indicators have been adequately measured and adjusted for.

Host exposure factors further complicate interpretation and may partly explain inconsistent microbial directions across studies. Smoking is a clear example. Mohammed et al. analyzed saliva from 200 smokers and 100 non-smokers in a Qatari population and reported that smoking markedly altered the salivary microbiome; *Streptococcus* was positively correlated with LDL and negatively correlated with HDL (59). Thus, lipid-associated microbial signatures in that study may partly reflect smoking-induced oral dysbiosis rather than dyslipidemia alone. Medication exposure is another important source of bias. Kaminska et al. showed that statins can affect multispecies oral biofilms and that simvastatin inhibits *P. gingivalis* growth (48), indicating that lipid-lowering therapy may reshape oral microbial communities while also modifying cardiometabolic risk. Antibacterial mouthwash can suppress nitrate-reducing bacteria, reduce nitrite production, and affect blood pressure regulation (86, 87, 90), meaning that mouthwash use and oral hygiene practices are not merely background variables but can directly influence GLMD-relevant functional pathways. Dietary exposure is also important: high-fat diet can remodel salivary and gut microbiota in animal models (60), whereas nitrate-rich foods can modify oral nitrate-reducing activity and NO-related vascular responses (88, 89). Recent antibiotics, proton pump inhibitors, antidiabetic drugs, lipid-lowering agents, antihypertensive drugs, and anti-inflammatory medications should therefore be recorded and controlled where possible, because they may affect either the oral microbiome, metabolic phenotype, or both.

In addition to biological confounding, methodological heterogeneity contributes substantially to the inconsistent directions. Taxonomic resolution is a major issue. For example, *Streptococcus* was reported as increased in oral swab samples from Chinese patients with T2DM (34), in tongue-coating samples from patients with MAFLD (55), and in salivary microbiota from high-fat diet-induced obese mice (60). However, *Streptococcus* was decreased in a salivary multi-omics study of childhood obesity/metabolic syndrome (81), while hypertension studies reported species-level associations involving *Streptococcus anginosus, Streptococcus salivarius*, and *Streptococcus lactarius* (50). These findings are not directly equivalent because genus-level *Streptococcus* contains species with different ecological niches, acidogenic capacity, nitrate-related functions, biofilm behavior, and inflammatory potential. Similar caution applies to broad genera such as *Prevotella, Veillonella, Haemophilus, Actinomyces, Aggregatibacter, Porphyromonas*, and *Fusobacterium*. Directional inconsistency at the genus level may therefore reflect species-level heterogeneity rather than true biological contradiction.

Sampling site is another major source of heterogeneity. Saliva reflects a mixed and relatively transient microbial signal, supragingival plaque captures biofilm-associated communities, subgingival plaque is strongly influenced by periodontal ecology, oral swabs combine signals from several mucosal surfaces, and tongue coating represents a distinct dorsal tongue ecosystem. Diabetes-related studies illustrate this point clearly. Li et al. analyzed both saliva and supragingival plaque from T2DM patients and reported enrichment of *Porphyromonas gingivalis* and *Prevotella melaninogenica* (52), whereas Chen et al. used oral swabs and found increased *Neisseria, Streptococcus, Haemophilus*, and *Pseudomonas* but decreased *Acinetobacter* (49). Wang et al. examined saliva from elderly Chinese individuals across fasting glucose categories and found enrichment of *Leptotrichia, Staphylococcus, Catonella*, and *Bulleidia* in the very-high fasting glucose group (47). These studies all address glucose dysregulation, but their sampled niches are not interchangeable. Therefore, inconsistent directions of taxa such as *Streptococcus*, *Prevotella, Haemophilus*, and *Leptotrichia* may partly reflect niche-specific microbial ecology rather than disease effects alone.

Analytical pipelines and evidence types further affect comparability. Chen et al. used oral swabs, V1-V2 16S rRNA sequencing, and QIIME in Chinese T2DM patients (49), whereas Wu et al. used V3-V4 16S sequencing of saliva in adults with obesity (45), Rahman et al. used nanopore-based 16S sequencing of subgingival plaque in overweight and obese individuals (53), and Lyu et al. used bidirectional Mendelian randomization rather than direct microbial sequencing (57). For dyslipidemia, Takagi et al. linked tongue-coating *Megasphaera* to dyslipidemia onset in a Japanese community-based study (61), whereas Prince et al. analyzed subgingival plaque in a South African cohort with metabolic syndrome and reported enrichment of *Actinomyces, Corynebacterium*, and *Fusobacterium* (51). These studies differ not only in sample type but also in sequencing region, taxonomic assignment, statistical modeling, endpoint definition, and whether the evidence concerns baseline association, incident disease, microbial transmission, or functional metabolism.

Taken together, the inconsistent directions in results do not refute a relationship between the oral microbiome and GLMD. Rather, they indicate that recurrent taxa should be interpreted as context-dependent microbial signals. *Streptococcus, Prevotella, Veillonella, Haemophilus, Neisseria, Porphyromonas, Fusobacterium, Aggregatibacter, Tannerella, Actinomyces, Granulicatella, Leptotrichia, Selenomonas, Megasphaera*, and *Bergeyella* repeatedly appear across GLMD-related studies, but their reported direction may be influenced by oral health status, host exposure, taxonomic level, oral niche, population background, disease stage, model system, sequencing platform, and analytical strategy. Future studies should therefore report periodontal status, dental caries and plaque indices, oral hygiene practices, mouthwash use, smoking and alcohol exposure, dietary information, recent antibiotics, proton pump inhibitors, antidiabetic, lipid-lowering, antihypertensive and anti-inflammatory medications, age, sex, ethnicity, and comorbidity profile. Standardized reporting frameworks for human microbiome studies, such as the STORMS checklist, may help improve transparency and comparability across cohorts (95). More importantly, analyses should distinguish oral-health-associated taxa, exposure-sensitive taxa, and metabolic-disease-associated taxa. Only microbial features that remain reproducible after controlling for these factors should be considered robust candidate biomarkers or mechanistic clues for GLMD.

#### 4.7.2 Causal inference and evidence boundaries in oral microbiome studies

After considering confounding and methodological heterogeneity, a separate question is whether observed oral microbial signatures have causal relevance to GLMD. Causal inference remains a central challenge because most human studies linking oral dysbiosis with metabolic disorders are observational. Cross-sectional studies can identify disease-associated microbial differences, but they cannot determine whether oral dysbiosis precedes metabolic dysfunction, results from metabolic disease, or reflects shared upstream factors such as periodontal inflammation, diet, medication exposure, or oral hygiene. Even prospective studies can establish temporal sequence but cannot fully eliminate residual confounding. Therefore, microbial differences observed in patients with GLMD should generally be interpreted as disease-associated signals rather than direct causal agents unless supported by longitudinal, interventional, genetic, or mechanistic evidence.

Different evidence types provide different levels of causal support. Mendelian randomization can reduce reverse causation and confounding, and has been applied to oral microbiome-metabolic disease questions, but its validity depends on robust microbial genetic instruments, adequate taxonomic resolution, population transferability, and control of horizontal pleiotropy (57). Animal transfer, gavage, and human microbiota-associated models can provide mechanistic support, as illustrated by studies of oral-gut microbial transmission in hypertension (40), but antibiotic pretreatment, artificial colonization, host-species differences, and simplified ecological conditions may exaggerate causal effects compared with chronic human disease settings. Tissue-based detection of oral microorganisms in blood, vascular plaques, or distant organs provides biological plausibility for microbial dissemination, but detection of microbial DNA or even viable bacteria does not by itself prove that these taxa initiate or drive GLMD-related pathology. Similarly, machine-learning models that distinguish disease groups or predict outcomes support biomarker feasibility, but classification accuracy does not establish mechanistic causality.

Accordingly, oral microbial taxa should currently be viewed as candidate contributors, modifiers, or biomarkers of GLMD-related pathways rather than established universal causal drivers. Stronger causal inference will require longitudinal cohorts with repeated oral sampling before disease onset, standardized measurement of oral health and host exposures, intervention studies that modify defined oral microbial functions, strain-level metagenomics to track microbial transmission, metabolomics to link taxa with functional pathways, and experimental validation of host-microbe mechanisms. Such studies are needed to determine whether specific oral microbial changes are causes, consequences, or amplifiers of GLMD progression.

## 5 Mechanisms linking oral microbial dysbiosis to GLMD

### 5.1 Taste perception

Oral microbial dysbiosis may impair taste perception through multiple interacting pathways. Dysbiotic oral communities can promote local inflammatory responses, increasing mediators such as TNF-α and IL-6, which may affect taste bud renewal and contribute to taste bud reduction. Microbial metabolites, including SCFAs and other bioactive products, may modulate taste receptor signaling, including T1R-mediated sweet/umami sensing and T2R-mediated bitter sensing. In parallel, tongue coating and oral biofilm formation may physically limit the interaction between tastants and taste buds. These changes can lead to reduced taste sensitivity, altered dietary preference, and increased consumption of unhealthy foods, thereby potentially contributing to glucolipid metabolic disorders (GLMD). The resulting metabolic disturbance may further exacerbate oral microbial imbalance, forming a bidirectional feedback loop.

**FIGURE 4:**
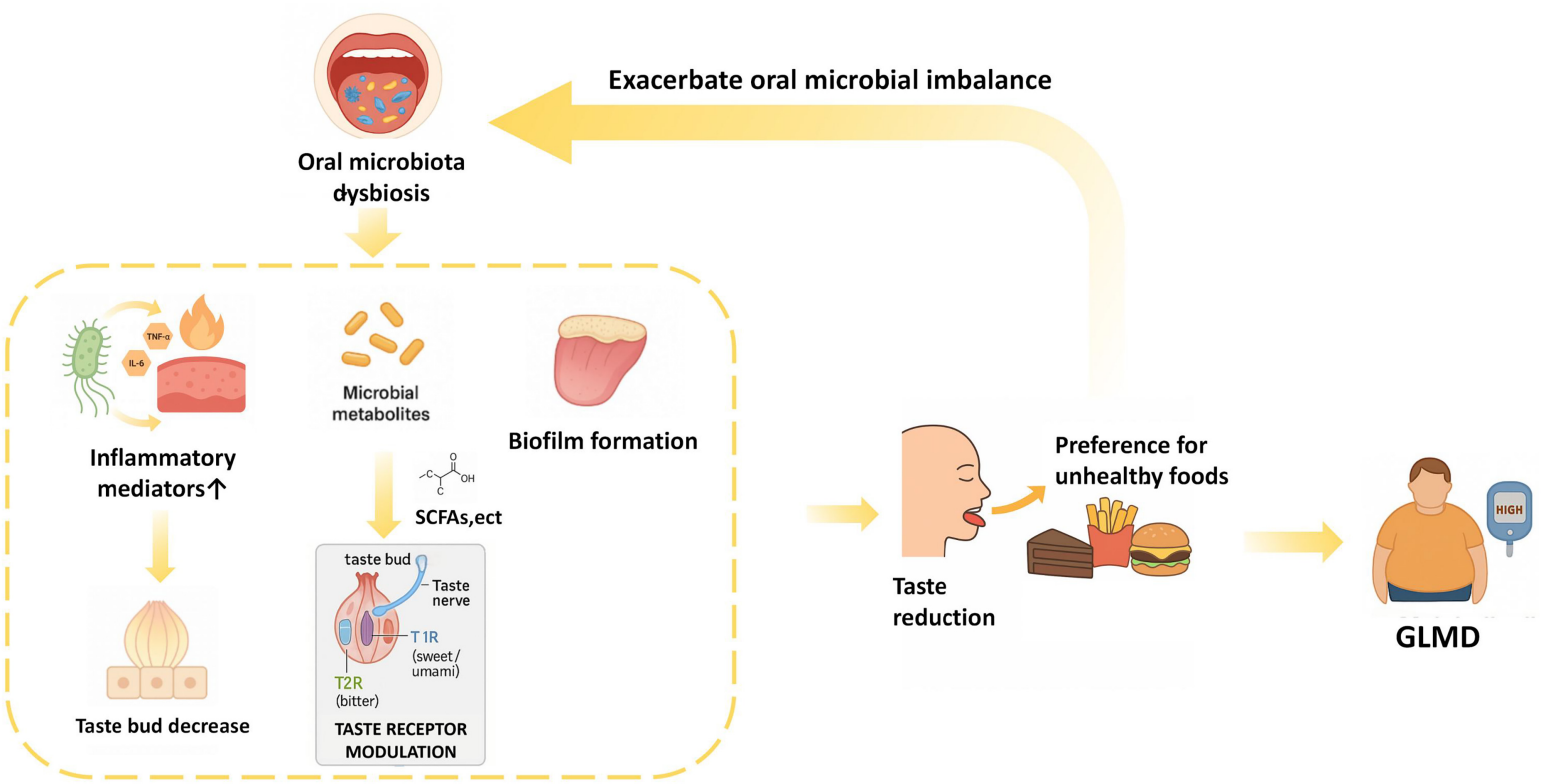
Impact of oral microbiota dysbiosis on taste perception.

Taste receptors mediate chemosensory perception and contribute to the regulation of food intake and metabolism, and they are not only located in the taste buds but also expressed in various oral and extraoral tissues. They can recognize the five basic taste modalities: sweet, salty, sour, umami, and bitter[95]. Sweet and umami stimuli are mainly detected by the type 1 taste receptors (T1Rs) heterodimer, and bitter compounds by type 2 taste receptors (T2Rs). Beyond detecting food flavor, these chemoreceptors play important roles in non-gustatory physiological and pathological processes. The changes in the microbial and inflammatory environment around taste papillae may influence GLMD not simply by changing food preference at the behavioral level, but by modifying receptor-level taste signaling (as illustrated in Figure 4).

Oral microbial communities may affect taste signaling through microbial ligands and epithelial immune sensing. Bitter taste receptors, especially T2Rs, are expressed not only in taste buds but also in gingival epithelial cells, solitary chemosensory cells, and other mucosal epithelial compartments. These receptors can detect bacterial products, including quorum-sensing molecules and other microbial metabolites, and activate innate defense responses involving Ca^2+^-dependent signaling, nitric oxide production, antimicrobial peptide release, and epithelial immune regulation (96). In this context, an enrichment of Gram-negative oral taxa such as *Porphyromonas, Fusobacterium, Prevotella*, and *Haemophilus* may be relevant because these bacteria can provide lipopolysaccharide (LPS), outer membrane vesicles, proteases, sulfur compounds, organic acids, and other microbial products that alter the local taste-papilla microenvironment. These products may act indirectly by changing pH, epithelial permeability, tastant availability, or inflammatory tone, rather than by serving as classical taste stimuli.

Inflammation provides a second molecular route linking oral dysbiosis to taste dysfunction. Microbial ligands such as LPS can activate pattern-recognition receptors, including TLR4/CD14 and TLR2, on epithelial and immune cells, leading to MyD88-dependent NF-κB and MAPK activation and subsequent production of TNF-α, IL-1β, and IL-6. These cytokines are biologically relevant to taste function because inflammatory stimulation has been shown to suppress taste progenitor cell proliferation and shorten the life span of taste bud cells (97, 98). More recent single-cell evidence further indicates that type II taste cells, particularly Tas1r3-expressing sweet/umami receptor cells, display mucosal immune-surveillance features. RANKL/TNFSF11 can promote M-cell-like programs in taste papillae through SPIB-related pathways, whereas SPIB deficiency alters NF-κB pathway components, cytokine responses, microbial transcytosis-like activity, and sweet/umami taste responses (99). These findings suggest that taste cells are not passive sensory targets but may actively integrate microbial and inflammatory signals.

The physical structure of the tongue coating and oral biofilm may further amplify these molecular effects. A thick or dysbiotic tongue coating can form a mechanical and biochemical barrier between tastants and taste pores, limiting the access of sugars, bitter compounds, amino acids, and fatty acids to their receptors. Biofilms enriched in anaerobic or inflammation-associated taxa may also generate local metabolites such as acetate, propionate, butyrate, volatile sulfur compounds, and proteolytic products. These metabolites may alter epithelial permeability, salivary redox status, and receptor accessibility. Consistent with this possibility, tongue cleaning has been reported to improve taste sensitivity (100, 101), and studies of tongue dorsum microbiota have linked specific bacterial taxa with sweet, salty, bitter, and sour taste thresholds (102).

These mechanisms are particularly relevant to GLMD because taste dysfunction may bias dietary selection toward energy-dense foods. In obese subjects, impaired orosensory lipid perception has been associated with specific gustatory papillae microbiota and salivary features. For example, lipid non-tasters showed distinct salivary and microbial signatures, and predicted bacterial pathways related to phosphotransferase systems and simple sugar transport were enriched in obese non-tasters (103). In insulin-resistant patients, defective fatty taste detection was associated with specific microbiota metabolism around the circumvallate papillae, while lower lipid taste sensitivity was more evident in patients treated with metformin and/or statins (104). After sleeve gastrectomy, patients with improved fat taste sensitivity showed a peri-circumvallate microbiota profile with lower *Porphyromonas, Fusobacterium*, and *Haemophilus* and higher *Atopobium* and *Prevotella* compared with patients without improvement (105). These findings support a plausible oral microbiota–taste receptor-dietary behavior axis in GLMD. However, because most human evidence remains associative, this pathway should be interpreted as a mechanistic hypothesis requiring direct experimental validation.

### 5.2 NO

Dietary nitrate is primarily found in vegetables, including celery, radishes, beets, and others. After ingestion, most dietary nitrate is absorbed in the gastrointestinal tract and enters the bloodstream, where it accumulates in the salivary glands via circulation. Sialin, a nitrate transporter protein in the salivary glands, actively mediates the uptake of up to 25% of circulating nitrate and concentrates it more than tenfold in saliva. This concentrated nitrate is then secreted into the oral cavity via saliva. Once in the mouth, nitrate-reducing bacteria convert salivary nitrate into nitrite. Upon swallowing, both nitrate and nitrite reach the stomach. In the acidic gastric environment, most nitrite is protonated to form nitrous acid (HNO2), which is subsequently reduced by antioxidants such as vitamin C and polyphenols to generate nitric oxide (NO) and other nitrogen oxides. The resulting NO exerts cardiovascular protective effects, including regulation of blood pressure and improvement of endothelial function.

**FIGURE 5:**
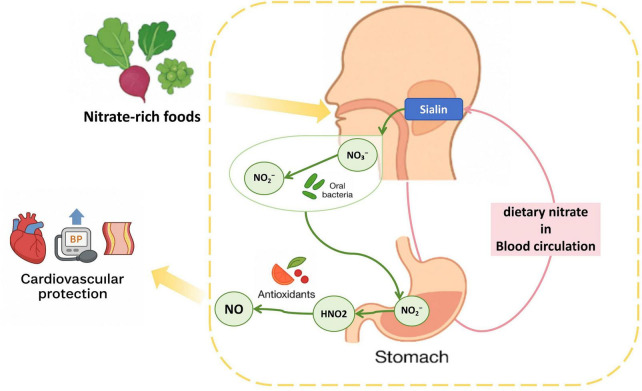
Oral microbiota mediated nitrate–nitrite–nitric oxide pathway.

NO is a gaseous signaling molecule that plays essential roles in vascular endothelial regulation, immune modulation, mitochondrial function, and neurotransmission. In the human body, NO is primarily generated through two distinct pathways (106). The first is the L-arginine–NO pathway, which depends on nitric oxide synthases (NOSs), including endothelial NOS (eNOS), neuronal NOS (nNOS), and inducible NOS (iNOS). The second is the nitrate-nitrite-nitric oxide pathway, and it is vital under hypoxic conditions. As illustrated in Figure 5, in this pathway, dietary nitrate (NO_3_^–^), mainly derived from green leafy vegetables, is concentrated via the bloodstream and secreted into the saliva. There, it is reduced by oral bacteria to nitrite (NO_2_^–^), which is subsequently reduced to NO under gastric acid conditions or in hypoxic tissue environments, thereby maintaining systemic NO homeostasis. The nitrate-nitrite-nitric oxide pathway thus represents a significant source of NO production and serves as an essential alternative to the classical NOS-dependent mechanism (107).

The oral microbiota is a crucial component of the nitrate-nitrite-nitric oxide metabolic axis. Nitrate-reducing bacteria mainly include thick-walled bacteria (*Staphylococcus*, *Streptococcus*, *Veillonella*), actinomycetes (*Rothia*, *Actinomyces*), Proteobacteria (*Neisseria*, *Haemophilus*, *Campylobacter*, *Pasteurella*), and Bacteroidetes (*Prevotella*) (108). At the molecular level, these taxa carry nitrate reductase systems, including membrane-bound or periplasmic nitrate reductases encoded by nar/nap-related genes, which catalyze the reduction of nitrate (NO_3_^–^) to nitrite (NO_2_^–^) (109). Species such as *Prevotella melaninogenica* and *Veillonella dispar* have been associated with higher salivary nitrite production after nitrate ingestion, indicating that the abundance and activity of nitrate-reducing taxa can shape oral nitrite generation (46).

The nitrite generated by oral bacteria can be swallowed and further reduced to NO through several routes. In the acidic gastric environment, nitrite is protonated to nitrous acid and decomposes to NO and other reactive nitrogen species. In blood and peripheral tissues, nitrite can also be reduced to NO by deoxyhemoglobin, deoxymyoglobin, xanthine oxidoreductase, mitochondrial enzymes, and other heme- or molybdenum-containing proteins, especially under hypoxic or acidic conditions (110). The generated NO activates soluble guanylate cyclase (sGC) in vascular smooth muscle cells, increasing cyclic guanosine monophosphate (cGMP) production and protein kinase G (PKG) signaling. This sGC-cGMP-PKG axis reduces intracellular Ca^2+^ availability, promotes myosin light-chain dephosphorylation, and induces vascular smooth muscle relaxation. NO also inhibits platelet aggregation, leukocyte adhesion, endothelial activation, and oxidative stress, thereby supporting endothelial homeostasis and vascular protection (110, 111).

This pathway is highly relevant to GLMD because impaired NO bioavailability is a shared feature of insulin resistance, obesity, hypertension, endothelial dysfunction, and atherosclerosis. Under physiological conditions, insulin activates the IRS-1/PI3K/Akt pathway in endothelial cells, leading to eNOS phosphorylation and NO production. In insulin-resistant states, this PI3K/Akt/eNOS branch is impaired, while MAPK-dependent vasoconstrictive and pro-inflammatory signaling may be relatively preserved, resulting in endothelial dysfunction and reduced microvascular perfusion. The oral nitrate-nitrite-NO pathway may partly compensate for this defect by providing an alternative source of NO that does not fully depend on eNOS activity (112). In experimental eNOS-deficient mice, dietary nitrate supplementation increased bioactive nitrogen oxides, reduced visceral fat accumulation and circulating triglycerides, and reversed prediabetic features, suggesting a mechanistic connection between nitrate-derived NO and systemic glucose-lipid homeostasis (112). In humans, concurrent beet juice and carbohydrate ingestion improved insulin sensitivity in obese adults when oral bacterial nitrate reduction was not inhibited; this effect was attenuated after antibacterial mouthwash, supporting a role for oral nitrate-reducing bacteria in postprandial metabolic regulation (113).

Oral microbial dysbiosis can disrupt this pathway at several points. A reduced abundance or activity of nitrate-reducing bacteria may lower salivary nitrite production and systemic NO bioavailability, whereas excessive use of antibacterial mouthwash can suppress oral nitrate reduction. In a randomized crossover study of treated hypertensive adults, 3 days of antibacterial mouthwash use reduced oral nitrate-to-nitrite conversion, decreased salivary nitrite, increased salivary nitrate, and raised systolic blood pressure (114). Similarly, case-control and intervention studies have linked lower levels of nitrate-reducing taxa, including *Neisseria* and *Veillonella*, with reduced salivary or plasma NO-related metabolites and higher blood pressure (86, 87). However, nitrate-reducing bacteria should not be interpreted simply as uniformly beneficial. Different taxa may participate in competing nitrate metabolic routes, and the effect of nitrate supplementation depends on oral ecological context, substrate availability, pH, redox state, diet, and oral hygiene. For example, inorganic nitrate has been proposed as a potential prebiotic-like modulator that can enrich health-associated nitrate-reducing genera such as *Neisseria* and *Rothia*, while reducing some acidogenic or periodontitis-associated bacteria in T2DM-related oral dysbiosis (115). The evidence is relatively strong for blood pressure regulation and endothelial function, whereas links with insulin resistance, obesity, dyslipidemia, and broader GLMD phenotypes still require larger longitudinal and interventional studies that jointly measure oral microbial composition, nitrate reductase activity, salivary/plasma nitrate-nitrite-NO metabolites, diet, medication exposure, and metabolic outcomes.

### 5.3 Inflammation

A decrease in nitrate-reducing bacteria and an increase in pathogenic bacteria (e.g., *P. gingivalis, Fusobacterium nucleatum, Aggregatibacter actinomycetemcomitans*) disrupt the oral barrier and lead to localized periodontal inflammation. Pathogen-associated molecular patterns such as lipopolysaccharide (LPS), outer membrane vesicles (OMVs), and bacterial proteases activate neutrophils and induce the release of inflammatory mediators. The impaired gingival epithelial barrier allows oral microbes and their products to enter the bloodstream, triggering activation of the NOD-like receptor thermal protein domain associated protein 3 (NLRP3) inflammasome and the generation of reactive oxygen species (ROS). These events can activate neutrophils, monocytes, and macrophages, which secrete proinflammatory cytokines, including interleukin-6 (IL-6), interleukin-1β (IL-1β), and tumor necrosis factor-α (TNF-α), thereby providing a plausible route to systemic inflammation relevant to GLMD.

**FIGURE 6:**
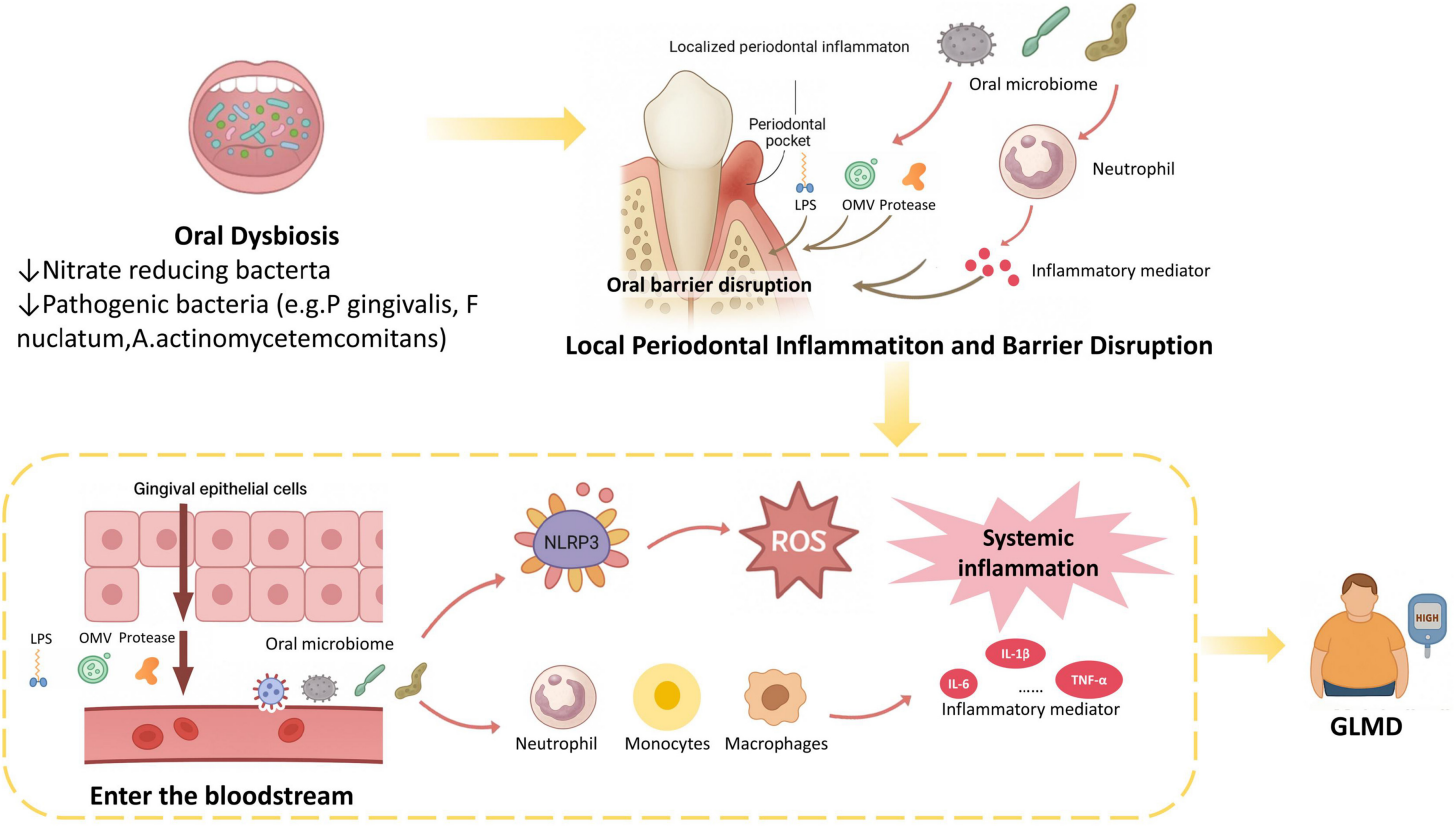
Mechanistic pathway linking oral dysbiosis to systemic chronic inflammation.

Systemic inflammation is recognized as a key mechanism linking dysbiosis of the oral microbiota to extraoral pathological processes. Oral bacteria and their metabolites can modulate host immunity through multiple routes (Figure 6): invading the circulation, releasing virulence factors, and activating innate immune receptors, resulting in chronic low grade inflammation that affects distant organs (116, 117). In a healthy oral ecosystem, commensal microbial communities interact with epithelial cells, neutrophils, macrophages, dendritic cells, and salivary antimicrobial factors to maintain barrier integrity and immune tolerance. When this balance is disrupted, polymicrobial communities enriched in pathobionts such as *Porphyromonas gingivalis*, *Fusobacterium nucleatum, Tannerella forsythia, Treponema denticola, Aggregatibacter actinomycetemcomitans*, and *Prevotella* species can promote a dysbiosis-inflammation feedback loop (118, 119). These organisms do not act only as isolated pathogens. Instead, they form spatially organized biofilms and exchange metabolic and chemical signals, which increase the collective inflammatory potential of the community (118). In susceptible hosts, this dysbiotic biofilm can impair epithelial barrier function, increase gingival permeability, and expose host cells to pathogen-associated molecular patterns (PAMPs), including lipopolysaccharide (LPS), lipoproteins, peptidoglycan, fimbriae, outer membrane vesicles (OMVs), gingipains, leukotoxin, and bacterial DNA.

At the receptor level, these microbial ligands activate pattern-recognition receptors and complement-related pathways. LPS from Gram-negative oral bacteria can bind LPS-binding protein and CD14, then activate TLR4/MD-2 signaling in epithelial cells, monocytes, macrophages, adipocytes, hepatocytes, and vascular endothelial cells. Bacterial lipoproteins, fimbriae, and peptidoglycan can also activate TLR2-dependent signaling. Downstream, MyD88-dependent and, in some contexts, TRIF-dependent signaling activates IRAK/TRAF6, IKKβ, NF-κB, and MAPK pathways, including p38, JNK, and ERK. These cascades induce transcription of IL-1β, IL-6, TNF-α, IL-8/CXCL8, CCL2/MCP-1, CRP-related inflammatory responses, and endothelial adhesion molecules such as ICAM-1 and VCAM-1 (92, 120). *P. gingivalis* may further amplify this response through gingipains and fimbrial proteins. *Gingipains* can degrade extracellular matrix and immune proteins, generate complement fragments, and alter neutrophil and macrophage responses, whereas FimA-related structures can interact with receptors such as TLR2, complement receptor 3 (CR3), and CXCR4 in oral-gut or systemic inflammatory contexts (121). In this way, oral dysbiosis may generate both local periodontal inflammation and low-grade systemic inflammatory signals relevant to GLMD.

Inflammasome activation provides another molecular bridge. Oral pathobionts and their products can prime inflammasome-related genes through TLR-NF-κB signaling and then promote assembly of the NLRP3 inflammasome in macrophages, epithelial cells, or vascular immune cells. This process involves NLRP3, ASC, and pro-caspase-1, leading to caspase-1 activation, cleavage of pro-IL-1β and pro-IL-18 into mature IL-1β and IL-18, and, in some settings, gasdermin D-mediated pyroptotic cell death. *P. gingivalis, F. nucleatum*, and *A. actinomycetemcomitans* have all been implicated in inflammasome-related periodontal and vascular inflammation (92, 120). The metabolic relevance is substantial: IL-1β can impair pancreatic β-cell function and insulin secretion, IL-18 participates in systemic inflammatory regulation, and persistent NLRP3 activation in adipose tissue, liver, and vascular tissues can contribute to insulin resistance, hepatic inflammation, endothelial activation, and atherosclerotic plaque instability. Therefore, the oral microbiome may be linked to GLMD through repeated delivery of inflammasome-activating microbial ligands and systemic propagation of IL-1β/IL-18-centered inflammation.

SCFA-mediated signaling should also be interpreted in a context-dependent manner. In the gut, acetate, propionate, and butyrate often support metabolic and immune homeostasis by activating FFAR2/GPR43, FFAR3/GPR41, and GPR109A, and by inhibiting histone deacetylases (HDACs), thereby promoting regulatory T-cell activity, IL-10 production, epithelial barrier function, and anti-inflammatory metabolic effects (122). However, in the periodontal pocket and dysbiotic oral biofilm, locally high concentrations of SCFAs, especially butyrate and propionate, may have different effects. These metabolites can alter epithelial barrier integrity, induce oxidative stress or apoptosis in gingival epithelial cells, and modulate IL-6, IL-8, TNF-α, and IL-1β production depending on concentration and inflammatory context (123). Therefore, SCFAs should not be described simply as beneficial or harmful. In GLMD, their effect may depend on anatomical site, local concentration, microbial source, receptor expression, host metabolic state, and whether the dominant pathway is FFAR-mediated immune regulation, HDAC inhibition, or epithelial injury.

Immune-metabolic cross-talk is further reinforced by adaptive immune responses. Hyperglycemia can increase IL-17 activity and alter oral microbial pathogenicity. In an experimental diabetes model, diabetes-enhanced IL-17 changed the oral microbiota and increased its pathogenicity; blockade of IL-17 reduced neutrophil recruitment, IL-6, RANKL expression, and bone resorption after transfer of oral microbiota to germ-free mice (124). This finding is important because it shows that systemic metabolic status can reshape oral immune-microbial interactions, while oral dysbiosis can in turn intensify local and systemic inflammatory responses. Recent human studies also support a relationship between oral microbiota, cytokine profiles, and cardiometabolic risk. For example, obesity-related hypertension has been associated with oral and gut microbial differences and correlations between microbial genera and pro-inflammatory cytokines (125), while salivary microbial indicators of periodontitis have been linked to cardiometabolic disease mortality in a national cohort (126). Taken together, current evidence supports an inflammation-centered oral microbiome-GLMD axis, and the strongest interpretation is that oral dysbiosis may amplify existing metabolic inflammation.

### 5.4 Translocation and colonization

Oral microbial dysbiosis may promote extraoral dissemination through two principal routes. First, disruption of oral epithelial or periodontal barriers can allow oral microorganisms and microbial products to enter the bloodstream. Second, swallowed oral microbes may reach the digestive tract, where gut dysbiosis and intestinal barrier dysfunction can facilitate further microbial translocation. These routes converge on the blood circulation route and may deliver oral taxa or microbial products to distant tissues. In the cardiovascular compartment, microbial signals may be associated with vascular plaques, cardiac tissues, and vascular inflammation, thereby potentially participating in processes related to cardiovascular disease, myocardial infarction, and arrhythmia. Through the portal venous route, oral-gut microbial transmission may expose the liver to microbial components and metabolites, which may be involved in hepatopathy and fibrosis.

**FIGURE 7:**
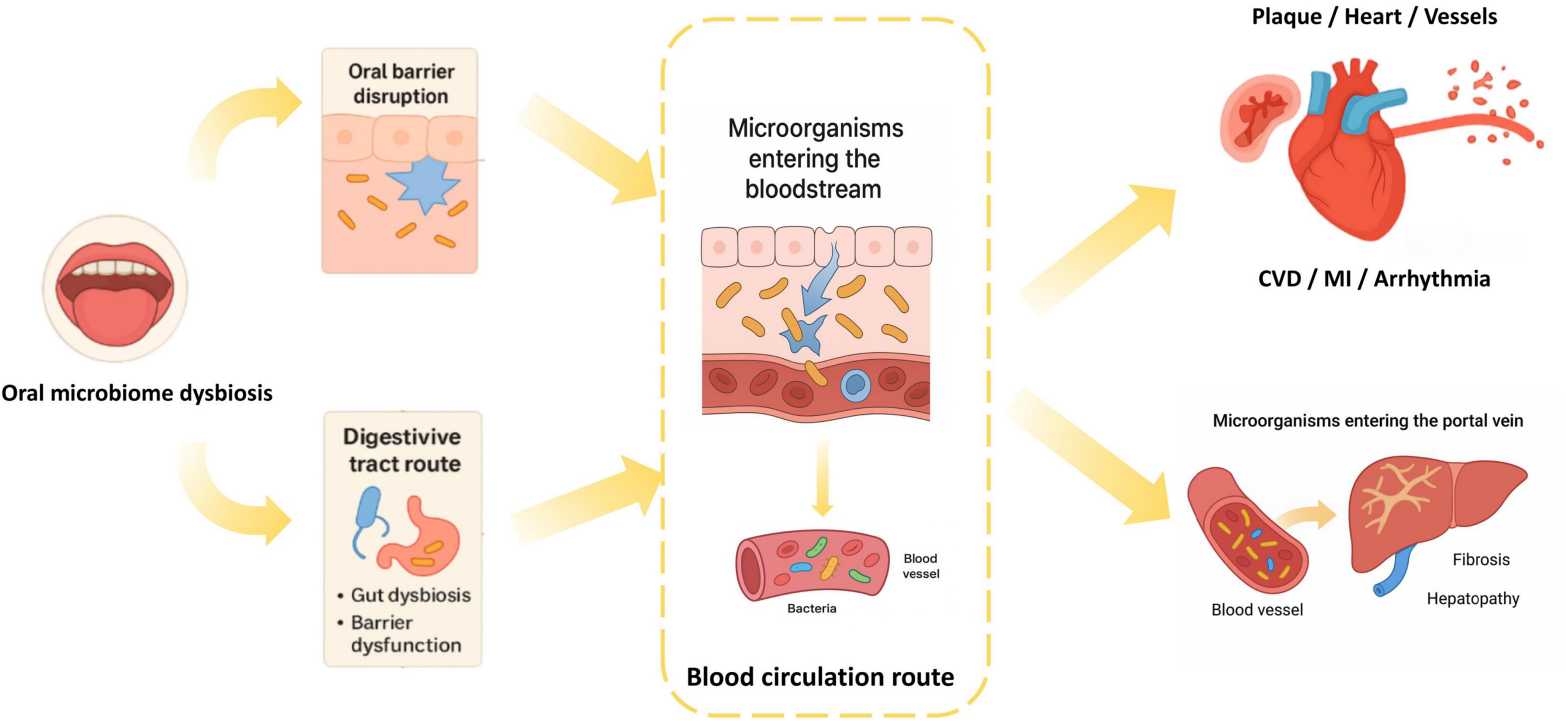
Ectopic colonization pathways of the oral microbiome.

Under certain conditions, oral microorganisms have been detected or proposed to translocate to distant body sites, where they may disturb local microbial ecology or immune homeostasis (Figure 7). The translocation and colonization of oral microbiota in the gut have long been recognized, and accumulating evidence now highlights the translocation of oral microbes into the gut, placenta, respiratory system, and bloodstream, which has been implicated in several chronic diseases, including cardiovascular diseases, cancer, and malnutrition (127).

Invasive dental procedures, oral mucosal ulcers, and periodontitis may provide entry routes for oral microorganisms and microbial products to cross the oral barrier and enter the circulation. Oral microbiota and their products can disseminate through the bloodstream and reach distant organs. Additionally, the gastrointestinal tract is a major route for oral microbial translocation, as approximately 1–2 L of saliva containing large numbers of microorganisms are swallowed daily.

Although most swallowed oral microorganisms are restricted by gastric acidity, bile acids, intestinal colonization resistance, and mucosal immunity, some taxa may survive under conditions of gut dysbiosis or barrier dysfunction and influence intestinal microbial networks or downstream translocation events (128). As previously discussed, oral microbiota can enter the bloodstream, migrate, and colonize vascular plaques, may be associated with impaired myocardial function and increased cardiovascular risk. In March 2025, Japanese researchers further showed in an experimental model that *P. gingivalis* can enter the heart via the bloodstream, thereby providing a possible mechanistic link with atrial fibrillation risk (129). Similarly, circulating oral microbial signatures have been associated with myocardial infarction, and experimental studies suggest that selected oral pathobionts may participate in post-infarction inflammatory responses (130). In August 2025, a Chinese research team reported in Circulation that pathogenic species such as *P. gingivalis*, *Fusobacterium nucleatum, Selenomonas sputigena, Prevotella intermedia*, and *Bacteroides plebeius* may disrupt epithelial and endothelial barriers and translocate into infarcted cardiac tissue. These oral pathobionts mobilized B cells, particularly B2 cells that produced interleukin-6 and tumor necrosis factor-α, to aggravate MI. Mechanistically, oral pathobiont-promoted B cells egress from cervical lymph nodes by a sphingosine-1-phosphate-sphingosine-1-phosphate receptor axis and infiltrate infarcted hearts by a C-X-C motif chemokine ligand 13-C-X-C motif chemokine receptor 5 axis (131).

Oral microbial translocation and possible ectopic colonization may also be involved in liver disease, particularly through the oral-gut-liver and portal circulation axes. Through disruption of the oral mucosa or the digestive tract, oral microorganisms can enter the portal circulation, increasing the microbiota in the bloodstream of hepatopathy patients. The circulating microbial components may be linked to alterations in the liver microbial or inflammatory milieu, thereby exacerbating clinical complications associated with liver disease (132). The concept of the “blood microbiota” in hepatic pathology was first introduced by the group of Jacques Amar, who reported a strong association between circulating microbiota and liver fibrosis in obese individuals. The blood microbiota of obese patients was dominated by *Proteobacteria*, with low concentrations of *Actinobacteria, Firmicutes*, and *Bacteroidetes* (133). Subsequent studies in patients with liver disease confirmed this finding, showing increased microbial diversity and a consistent predominance of *Proteobacteria* in the bloodstream, with low concentrations of *Actinobacteria, Firmicutes*, and *Bacteroidetes* (132). Specific taxa, such as *Sutterellaceae*, *Dyella*, and *Gemmatimonas*, may migrate along the oral–gut axis and show a positive correlation with the severity of alcoholic liver disease (134).

The mechanisms by which translocated oral microorganisms influence disease development remain under investigation. Inflammation, particularly chronic inflammation, has been recognized as a key mediator linking oral microbes to both local and systemic health effects. Microbial translocation from the oral cavity can disrupt immune homeostasis and compete with resident microbiota for ecological niches. Major microbial products and metabolites, including SCFAs, TMAO, indole and its derivatives, bile acids, and LPS, may affect inflammation, oxidative stress, lipid and glucose metabolism, and the integrity of biological barriers (121). However, the taxon-specific mechanisms, molecular targets, viability of translocated microorganisms, and causal relevance of distant microbial signals require further clarification before these pathways can be considered established drivers of GLMD progression.

## 6 Prevention and treatment

In recent years, interventions targeting the oral microbiota have shown therapeutic potential in metabolic diseases. Approaches such as periodontal therapy (e.g., scaling and root planning combined with antibiotics), enhanced oral hygiene, dietary interventions (e.g., dietary nitrate, green tea kombucha), exercise, and traditional Chinese medicine formulations (e.g., Artesunate, Guanxinning, Huoxue Jiangtang Decoction) have been reported to modulate oral microbial communities. Some studies reported reduced pathogen abundance, enrichment of health-associated taxa, and improvements in selected metabolic or inflammatory indicators (see Table 2 for details).

**TABLE 2:** Efficacy of oral microbiota-targeted interventions on metabolic diseases.

Evidence level/study type	Intervention category	Method/agent	Disease treated	Study subject (population/model, n)	Intervention method	Relevant oral microbiota	Main benefits
Human clinical study	Periodontal treatment/Dietary nitrate intervention	Periodontal treatment and nitrate-rich beetroot juice (BRJ)	Periodontitis-associated impairment of nitrate-mediated blood pressure regulation	Human adults, *n* = 30 total; periodontitis patients, *n* = 15; periodontally healthy controls, *n* = 15	Periodontitis group: PMPR and oral hygiene instruction and subgingival instrumentation; no antibiotics/antiseptics. Healthy control : no periodontal treatment. BRJ: 350 mL, 4.23 g/L nitrate; BP/saliva measured pre- and 1.5 h post-BRJ. Follow-up: 70 days	Subgingival plaque and tongue coating: Nitrate-reducing bacteria, *Rothia, Veillonella, Actinomyces*, and *Kingella;* periodontitis-associated bacteria including *P. gingivalis, Filifactor alocis, Tannerella forsythia, Prevotella intermedia*, and *Fretibacterium*	BRJ significantly reduced SBP and DBP in healthy individuals, but not in untreated periodontitis patients. After periodontal treatment, the BP-lowering response to BRJ was restored; periodontitis-associated bacteria decreased, and nitrate-reducing bacteria increased in subgingival plaque (137)
Human clinical study	Periodontal treatment and systemic anti-inflammatory therapy	Periodontal treatment and systemic anti-inflammatory therapy	Stage III-IV periodontitis and T2DM	Human patients (T2DM and Periodontitis, *n* = 32 total; Test Group *n* = 17;Control Group *n* = 15)	Test Group: Full-mouth SRP and Oral antibiotics (AMX and MTZ, TID for 7 days); Control Group: Full-mouth SRP only, Follow-up: 3 months.	Subgingival plaque: Red complex, *P. gingivalis, T. forsythus, Treponema, Porphyromonas, Capnocytophaga, PeptostreptococcaceaeXIG-6, Neisseria, Pseudomonas, Saccharibacteria TM7 G-5, Actinomyces*	Test Group showed: Reduced HbA1c and glucose; greater reduction in periodontal pathogens (“red complex”), greater improvement in clinical parameters (PD, AL, BI), greater reduction in subgingival dysbiosis index (SMDI), and greater improvement in systemic inflammatory markers (serum IL-17, OPG/RANKL ratio) (135)
Human clinical study	Periodontal treatment	Scaling and root planning (SRP)	Chronic periodontitis with and without T2DM	Human patients (*n* = 22 total; *n* = 11 CP only; *n* = 11 CP and T2DM)	Full-mouth nonsurgical periodontal therapy (Scaling and Root Planing). Follow-up: 3 months	Unstimulated saliva and subgingival plaque: *Porphyromonas, Filifactor, Eubacterium* *Selenomonas, Actinobacteria, Streptococcus, Veillonella, Actinomyces, Leptotrichia, Prevotella*	Improved glycemic control (FBG, HbA1c) in the T2DM group. Increased microbial diversity and proportion of health-associated bacteria in the T2DM group. Microbial community networks became more complex and stable post-treatment in the T2DM group. Improved periodontal parameters (PD, CAL) in both groups. (136)
Human dietary intervention	Dietary interventions	Dietary nitrate (beetroot juice)	Hypertension	pregnant and nonpregnant women, with or without hypertension (*n* = 55 total; nonpregnant normotensive, *n* = 15, nonpregnant hypertensive, *n* = 7, pregnant normotensive, *n* = 21, pregnant hypertensive, *n* = 12)	Single dose of 400 mg nitrate in 70 mL beetroot juice after an overnight fast	Tongue scraping samples: *Veillonella*	Lower baseline DBP and greater DBP reduction with higher oral NaR activity; reduced salivary nitrite in hypertensive women (88)
Human dietary intervention	Dietary interventions	Green tea kombucha (GTK)	Excess body weight	Adults with BMI ≥ 27 kg/m^2^, circumference ≥ 80 cm for women and ≥ 94 cm for men, DEXA > 30% for women and > 25% for men (*n* = 65 total; CG, *n* = 29 analyzed, KG, *n* = 30 analyzed)	Control: caloric restriction (reduce 500 kcal). Kombucha: caloric restriction and 200 mL GTK. For 10 weeks.	Unstimulated saliva: *Catonella morbi, Schaalia odontolytica, Lachnoanaerobaculum umeaense, Eubacterium sulci, Megasphaera micronuciformis, Veillonella dispar, Oribacterium sinus, Prevotella pallens, Lancefieldella parvula, Bacillota, Firmicutes/Bacteroidetes*	Weight, BMI, Body fat decreased, Lipid accumulation product (LAP) reduced; prevented IL-6 increase; improved salivary alpha and beta diversity (138)
Human lifestyle intervention	Exercise interventions	Supervised exercise program	Non-alcoholic fatty liver disease	Obese middle-aged men with NAFLD and periodontal disease (*n* = 70 total: Exercise = 49, Diet = 21)	E group: 90 min exercise (aerobic and resistance) 3 times/week for 12 weeks. D group: 90 min dietary restriction program lecture or a consultation, 1 times/week for 12 weeks.	Saliva: *Campylobacter, Corynebacterium, Actinomyces, Lautropia, Prevotella*	Reduced fat mass and increased lean body mass; Salivary LPS and lactoferrin reduced; improved oral bacterial diversity; reduced LPS biosynthesis genes (75)
Preclinical animal study	Systemic anti-inflammatory therapy	Artemisia annual	Diabetic xerostomia in T2DM	Male SD rats (T2DM model, *n* = 40 total; *n* = 8/group)	ART groups: ART (10 mg/kg or 50 mg/kg) once daily for 4 weeks. Met groups: Met (150 mg/kg) once daily for 4 weeks. Con and Dia groups: equivalent amounts of the saline.	Rat oral swabs: *Veillonella, Lactobacillus, Clostridium sensu stricto 1, Escherichia-Shigella, Dubosiella, unidentified_Chloroplast, c Mycoplasma, Arsenophonus, Gemella, Proteus, Muribacter, Rothia, Streptococcus, Staphylococcus*	Reduced hyperglycemia and insulin resistance, improved saliva flow, ameliorated salivary gland injury, restored oral microbiota and metabolic profiles, suppressed NF-κB/NLRP3 pathway (143)
Preclinical mechanistic study in animal model	Traditional Chinese Medicine	GXN extract powder	Diabetic myocardial ischemia-reperfusion Injury	Diabetes model (Exp. 1 *n* = 4–6/group, Exp .2 *n* = 5–8/group, Exp. 3 *n* = 5–11/group)	Experiment 1: CON, MIRI, DMIRI groups, equivalent volume of water daily; Dapa group, 10 mg/kg of dapagliflozin per body weight daily; GXN-H and GXN-L, 1,200 mg/kg and 600 mg/kg of GXN extract powder, for 6 weeks Experiment 2: antibiotic gavage plus the same medication regimen as in Experiment 1, for 6 weeks Experiment 3: Gavage was administered for 6 weeks, with every other day administration for the first 2 weeks, followed by once a week for the next 4 weeks.	Oral swabs and colonic contents: *Fusobacterium nucleatum* (oral)	Reduced myocardial infarct size, decreased cardiomyocyte apoptosis, lowered serum cTnI, modulated oral F. nucleatum abundance, regulated PTEN/PI3K signaling pathway (144)
Preclinical animal study	Traditional Chinese Medicine	Huoxue Jiangtang Decoction	T2DM	Male SD rats (HFD/STZ-induced T2DM model, *n* = 32 total; *n* = 8/group)	HJD (15.86 g/kg/day), or metformin (0.158 g/kg/day), or 2 mL of saline (0.9%), for 8 weeks	Saliva: *Firmicutes, Proteobacteria, Actinobacteria, Streptococcus, Lactobacillus, Veillonella, Rothia, Pseudomonas, Negativicutes, Selenomonadales, Bifidobacterium*	Improved FBG, glucose tolerance, lipid profile; reduced preferences for fat and sugar; modulated oral microbiota; enhanced β-cell function (145)
Preclinical animal study	Oral hygiene	0.2% Chlorhexidine mouthwash	Diet-induced obesity and associated metabolic dysfunctions	Round 1: 24 mice, two groups (*n* = 12 each), Standard Rodent Diet (RD). Round 2: 24 mice, two groups (*n* = 12 each), Western Diet (WD)	Round 1: Chlorhexidine mouthwash vs. Saline mouthwash, 8 weeks. Round 2: Chlorhexidine mouthwash vs. Saline mouthwash, 8 weeks.	Oral cavity swabs, cecal contents and fecal samples: *Lactobacillus, Faecalibaculum, Clostridiaceae 1* and *Atopobiaceae families, Eubacterium coprostanoligenes, Peptococcaceae, Oscillibacter, Ruminiclostridium*	Reduced weight gain, fat mass, hepatic steatosis, plasma insulin; Impaired nutrient absorption (increased fecal triglycerides/protein);Reduced oral and gut bacterial richness; Altered gut microbiota (17)

Available human clinical and intervention studies provide preliminary support for the clinical relevance of oral microbiota-targeted strategies. Periodontal therapy, including scaling and root planning with or without systemic antibiotics, reduced periodontal pathogens and inflammatory markers and was associated with improved glycemic indicators in patients with T2DM and periodontitis (135, 136). From a nutritional perspective, nitrate-rich beetroot juice represents a key example linking oral nitrate-reducing bacteria, such as *Veillonella*, to blood pressure regulation. Sanchez-Orozco et al. reported that the blood pressure-lowering response to nitrate-rich beetroot juice was impaired in untreated periodontitis patients but restored after periodontal treatment, accompanied by reduced periodontitis-associated taxa and increased nitrate-reducing bacteria in subgingival plaque (137). Other dietary and lifestyle interventions, including beetroot juice, green tea kombucha, and supervised exercise, also improved oral microbial profiles and metabolic or inflammatory indicators in populations with hypertension, excess body weight, or NAFLD (75, 88, 138).

Beyond direct interventions, several emerging research directions offer new strategies for the prevention and management of metabolic diseases. Engineered oral microbiota based on synthetic biology principles can deliver antioxidant enzymes, anti-inflammatory cytokines, and metabolic-regulating peptides such as Glucagon-Like Peptide-1 (GLP-1) in the oral cavity, opening new avenues for precise intervention in cardiovascular and metabolic disorders (139). Systemic management of periodontitis (140), targeting specific detrimental microbial metabolites or signaling pathways (91), suppression of virulence factors, application of natural compounds (e.g., carnosic acid) and RNA interference technologies, as well as modulation of the oral–gut axis through prebiotics, probiotics, and microbiota transplantation (141), all show potential to improve metabolic conditions. Dietary adjustments, such as reducing sugar intake and supplementing with inorganic nitrate (115, 142), can also improve both the microbiota and metabolic indicators.

In the future, the oral microbiota may be considered as part of treatment strategies for GLMD. Before this can be implemented, Future work should clarify the mechanisms of key functional microbial taxa and pay close attention to the long-term safety and personalized use of microbial modulators. Understanding how these functional microbes influence disease processes may support the development of new targeted probiotics, dietary supplements, and traditional Chinese medicine formulations, and help shift from conventional “disease treatment” to an “ecological regulation” approach for the comprehensive management of GLMD.

## 7 Potential in disease prediction and therapeutic decision-making

The oral microbiome is accessible and can be sampled non-invasively for repeated sampling and population-level screening, however, clinical translation depends on whether these microbial signals remain reproducible across sampling sites, cohorts, sequencing platforms, analytical pipelines, and oral health backgrounds.

Evidence from non-metabolic diseases provides useful examples of both the promise and the limitations of oral microbiome-based prediction. In gastric cancer, Yuan et al. reported that tongue coating microbiome-based models achieved AUC values of 0.94 at the genus level and 0.95 at the species level, while the larger prospective multicenter and external validation stages mainly focused on tongue image-based models rather than microbiome-only models (146). This distinction is important because excellent performance in an initial microbiome cohort does not necessarily indicate that the same microbial model has been fully validated across centers. In colorectal cancer, Zhang et al. developed oral swab-based microbial classifiers and validated them in discovery and validation cohorts, but the authors noted that microbial biomarkers may be ethnicity-dependent and that using only oral swabs may introduce bias because tongue coating, saliva, oral wash, and swabs capture different oral ecological niches (147). Similarly, a prospective multicenter study of indeterminate pulmonary nodules compared saliva, throat swabs, and tongue coating samples and found saliva to have the best predictive performance, but the study also showed strong site-specific microbial differences and acknowledged the need for broader populations, more clinical variables, and larger malignant nodule cohorts (148). For digestive system tumors, a tongue coating microbiota-based XGBoost model achieved an AUC of 0.926 in internal validation, but the study was conducted in an eastern Chinese population, included relatively small numbers of hepatobiliary and pancreatic tumors, and required further validation in larger prospective cohorts (149). These studies support the feasibility of oral microbiome-based prediction, but they also show that sample type, population background, tumor spectrum, and validation design strongly influence model reproducibility.

In metabolic diseases, the available prediction evidence remains more preliminary. Liu et al. constructed a salivary microbiome-based auxiliary diagnostic model for T2DM using samples from 24 treatment-naive T2DM patients and 21 healthy controls, achieving an accuracy of 80% (150). This study is valuable because treatment-naive patients reduce medication-related confounding, but its cross-sectional design, small sample size, saliva-only sampling, and lack of external validation limit direct clinical translation. The authors also noted that small sample size and baseline oral health status may affect conclusions regarding salivary microbial alterations. By contrast, the Suzhou Cardiometabolic Health Study is an important prospective cohort protocol that plans to integrate tongue coating microbiota with metabolic profiling for subclinical target organ damage and cardiovascular risk stratification; however, it remains a study protocol, and outcome-based validation is still pending (151).

In addition to disease prediction, the oral microbiome may provide a means of selecting individualized therapeutic strategies. Changes in the salivary microbiome, for instance, can differentiate treatment responses to chemoradiotherapy in oral cancer patients (152). The tongue coating microbiome has emerged as a predictive biomarker for the efficacy of washed microbiota transplantation (WMT) in children with autism spectrum disorder (ASD). A predictive model integrating the tongue microbiome with clinical features achieved an accuracy of 73%, comparable to that of gut microbiota-based models (153). Given the convenience and non-invasive nature of tongue coating sampling, particularly for populations where fecal collection is challenging, it holds value for clinical application.

The oral microbiome is being explored as a potential source of complementary biomarkers. As previously discussed, there is a significant interplay between the oral and gut microbiota. Currently, the relationship between individual variability in drug response (IVDR) and the gut microbiome has been recognized (154), indicating that microbial biomarkers can inform personalized treatment decisions (155). For instance, specific statins can be selected based on the correlation between gut microbiota composition and drug response (156), and precision therapeutic strategies for cardiovascular disease patients can be tailored to gut microbiota characteristics (157). Owing to the greater convenience of sample collection and patient acceptability, the oral microbiome has become an alternative to fecal microbiota. The tongue coating microbiome study by Chen et al. provides a particularly relevant example for cardiometabolic intervention. In 94 healthy individuals, tongue coating microbial composition and predicted NO-producing capacity were associated with cardiovascular health indicators, and a chlorhexidine mouthwash intervention produced different blood pressure responses in participants with different tongue-coating phenotypes (90). This finding suggests that tongue coating microbiota and nitrate-reducing capacity may help stratify responses to oral ecological or nitrate-NO-related interventions. Nevertheless, the intervention was conducted in a small subgroup of self-reported healthy individuals rather than GLMD patients, and the study did not establish clinically validated microbial thresholds for selecting antihypertensive strategies. Therefore, it should be interpreted as proof-of-concept evidence rather than as a ready-to-use therapeutic decision tool.

A critical consideration is selecting and standardizing the optimal sampling site. Oral microbial profiles differ substantially among saliva, tongue coating, supragingival plaque, subgingival plaque, throat swabs, oral washes, and mucosal swabs, and these sample types should not be treated as interchangeable. For example, the indeterminate pulmonary nodule study directly compared saliva, throat swabs, and tongue coating samples and found clear site-specific microbial clustering, with saliva showing the best predictive performance in that setting (148). Similarly, the colorectal cancer study used only oral swabs and acknowledged that this could introduce bias because oral microbes are unevenly distributed across oral niches (147). Temporal stability is another unresolved issue. Following dental cleaning, plaque removal can markedly alter local microbial diversity, whereas saliva appears relatively more stable in some settings (158). However, stability after routine oral hygiene, dietary changes, mouthwash use, antibiotics, smoking, periodontal inflammation, proton pump inhibitors, and metabolic medication exposure remains insufficiently characterized. Therefore, future studies should report and control pre-analytical variables according to standardized microbiome reporting frameworks and should evaluate whether single time-point samples are sufficient for long-term risk prediction (95).

Taken together, the oral microbiome is a promising source of non-invasive candidate biomarkers, but clinical implementation requires a higher level of evidence than association or internally validated machine learning performance. Future studies should establish standardized protocols for sampling, storage, DNA extraction, sequencing, and bioinformatic analysis, because technical variation and analytical choices can substantially affect microbiome results (159–161). Predictive models should be externally validated in independent multicenter cohorts and calibrated against established clinical indicators. Clinically meaningful thresholds, sensitivity, specificity, positive and negative predictive values, and incremental value beyond conventional risk factors should be reported. For GLMD, particular attention should be paid to periodontal status, oral hygiene, smoking, diet, medication use, age, sex, ethnicity, and metabolic comorbidity, because these factors may independently influence both oral microbial composition and metabolic risk. Only after reproducible signatures, validated diagnostic thresholds, and prospective evidence of clinical utility are established can oral microbiome-based testing move from exploratory biomarker research toward practical risk stratification and individualized intervention.

## 8 Knowledge gaps and future directions

The application of high-throughput sequencing technology and omics analysis has significantly advanced our understanding of the complex and dynamic relationship between the oral microbiota and GLMD. However, substantial knowledge gaps and limitations persist in current research. The following points outline critical questions that need to be solved.

- Oral microbiome dysbiosis has been associated with GLMD-related conditions. However, when microbial dysregulation occurs, some patients develop metabolic comorbidities, while others exhibit only a single metabolic disorder. It remains unclear which individual patient factors influence susceptibility to single metabolic disorders or their co-occurrence. Identifying these susceptibility factors may help identify strategies to prevent single metabolic disorders from progressing to comorbid conditions, thereby reducing the burden of multiple metabolic dysfunctions.
- The bidirectional association between oral microbiome dysbiosis and GLMD has been observed, but the specific causal relationship remains unclear. Future research should prioritize rigorously designed, large-scale longitudinal cohort studies with long-term follow-up. These studies can collect data on disease incidence and risk to explore the strengthen causal inference between the oral microbiome and GLMD.
- Differences in ecological niches lead to variations in the structure of the oral microbiome. Prior research predominantly focused on oral diseases, specifically examining microbial communities associated with periodontitis and dental plaque. Subsequent research has increasingly focused on the oral microbiome itself in non-oral disease states, concentrating on samples from various niches such as saliva and dorsal tongue swabs. However, there remains a lack of systematic research on the link between the oral microbiota from these distinct niches and disease. Future studies should identify stable and representative oral niches to support follow-up studies.
- Compared to mice, the composition of the microbiome varies significantly across different human body regions, particularly in terms of bacterial colonization patterns, microbial composition changes, and host-microbe interactions (127). These differences suggest that animal models such as mice cannot fully reflect the complex microbiological characteristics and physiological environment of humans. Therefore, future research should focus on human sample studies. This includes long-term longitudinal cohort studies that integrate diverse human characteristics (e.g., age, sex, dietary patterns, geographical location, genetic background, among others) to establish the boundary between normal and dysbiosis states of the human oral microbiome, as well as the critical transition points. Such studies could clarify the structure and temporal dynamics of the human oral microbiome and its relationship with systemic metabolism.
- Research on the oral microbiome currently lacks standards for niche selection, sample collection, preservation methods, microbial detection, and data analysis. In the future, standardized procedures for sampling, sequencing, and bioinformatics analysis should be developed to enhance the comparability and reproducibility of results across different studies.

## 9 Conclusion

With the increasing global burden of metabolic diseases, multiple metabolic disorders have become a major global public-health challenge. Recent advances in research have revealed the link between the oral microbiome and metabolic disorders. The oral microbiome’s role in GLMD extends beyond merely reflecting the host state, and it may participate in disease-related processes through multiple mechanisms, including the modulation of taste perception, interference with NO production, induction of inflammatory responses, and promotion of bacterial translocation and ectopic colonization. A deeper exploration of these mechanisms may provide new biological insights and contribute to a comprehensive understanding of metabolic comorbidities. Therapeutic strategies targeting oral microecology may provide a complementary approach to conventional metabolic disease management, shifting from the traditional model of “disease treatment” to “ecological modulation.” Meanwhile, characteristic changes of the oral microbiome may become candidate biomarkers after further validation for disease prediction, risk assessment, and individualized intervention. Current evidence supports continued investigation of the oral microbiome as a source of mechanistic insights and candidate biomarkers, while clinical application will require standardized methods, external validation, and prospective evidence of utility.

## Footnotes

1
https://homd.org/
